# Widespread Family of NAD^+^-Dependent Sulfoquinovosidases at the Gateway to Sulfoquinovose Catabolism

**DOI:** 10.1021/jacs.3c11126

**Published:** 2023-12-15

**Authors:** Arashdeep Kaur, Isabelle B. Pickles, Mahima Sharma, Niccolay Madeido Soler, Nichollas E. Scott, Sacha J. Pidot, Ethan D. Goddard-Borger, Gideon J. Davies, Spencer J. Williams

**Affiliations:** † School of Chemistry, University of Melbourne, Parkville, Victoria 3010, Australia; ‡ Bio21 Molecular Science and Biotechnology Institute, University of Melbourne, Parkville, Victoria 3010, Australia; § York Structural Biology Laboratory, Department of Chemistry, University of York, York YO10 5DD, U.K.; ∥ ACRF Chemical Biology Division, The Walter and Eliza Hall Institute of Medical Research, Parkville, Victoria 3010, Australia; ⊥ Department of Medical Biology, University of Melbourne, Parkville, Victoria 3010, Australia; # Department of Microbiology and Immunology, University of Melbourne at the Peter Doherty Institute for Infection and Immunity, Melbourne, Victoria 3000, Australia

## Abstract

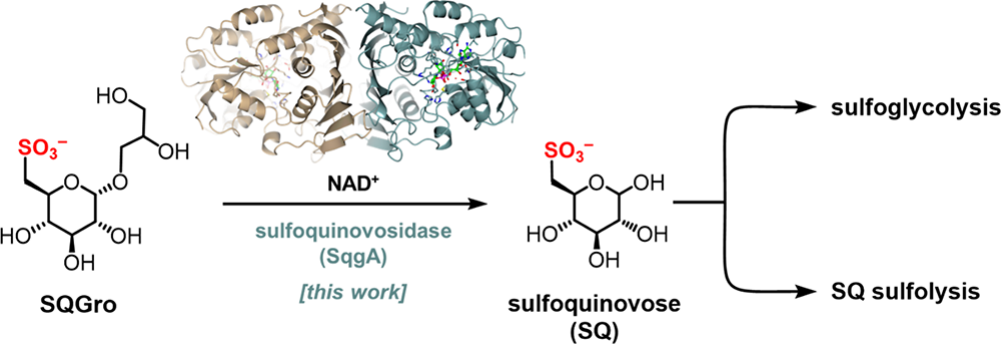

The sulfosugar sulfoquinovose (SQ) is produced by photosynthetic plants, algae, and cyanobacteria on a scale of 10 billion tons per annum. Its degradation, which is essential to allow cycling of its constituent carbon and sulfur, involves specialized glycosidases termed sulfoquinovosidases (SQases), which release SQ from sulfolipid glycoconjugates, so SQ can enter catabolism pathways. However, many SQ catabolic gene clusters lack a gene encoding a classical SQase. Here, we report the discovery of a new family of SQases that use an atypical oxidoreductive mechanism involving NAD^+^ as a catalytic cofactor. Three-dimensional X-ray structures of complexes with SQ and NAD^+^ provide insight into the catalytic mechanism, which involves transient oxidation at C3. Bioinformatic survey reveals this new family of NAD^+^-dependent SQases occurs within sulfoglycolytic and sulfolytic gene clusters that lack classical SQases and is distributed widely including within *Roseobacter* clade bacteria, suggesting an important contribution to marine sulfur cycling.

## Introduction

Sulfur is essential for life, yet significant gaps remain in our understanding of sulfur cycling through the biosphere.^1,2^ These knowledge gaps are inspiring renewed efforts to discover and characterize the biochemical pathways that mineralize abundant, but poorly studied, organosulfur compounds. The new pathways provide archetypes that allow annotation of the dark matter of environmental metagenomic data sets and illuminate the pathways of sulfur utilization by microbial communities in diverse natural environments. Sulfoquinovose (SQ; 6-deoxy-6-sulfoglucose) is one such abundant but neglected organosulfur compound that has attracted significant attention in recent years. It forms the headgroup of plant and cyanobacterial sulfolipid, sulfoquinovosyl diacylglycerol,^3^ and it is estimated that 10 billion tons of SQ are biosynthesized and degraded each year.^4^ As such, it is a key species in the biogeochemical sulfur cycle. Microbial degradation of SQ occurs in all environments in which plant matter is found: by soil and marine bacteria,^5,6^ and even enteric bacteria within the gastrointestinal tract of herbivorous and omnivorous animals.^7−9^

**Figure 1:**
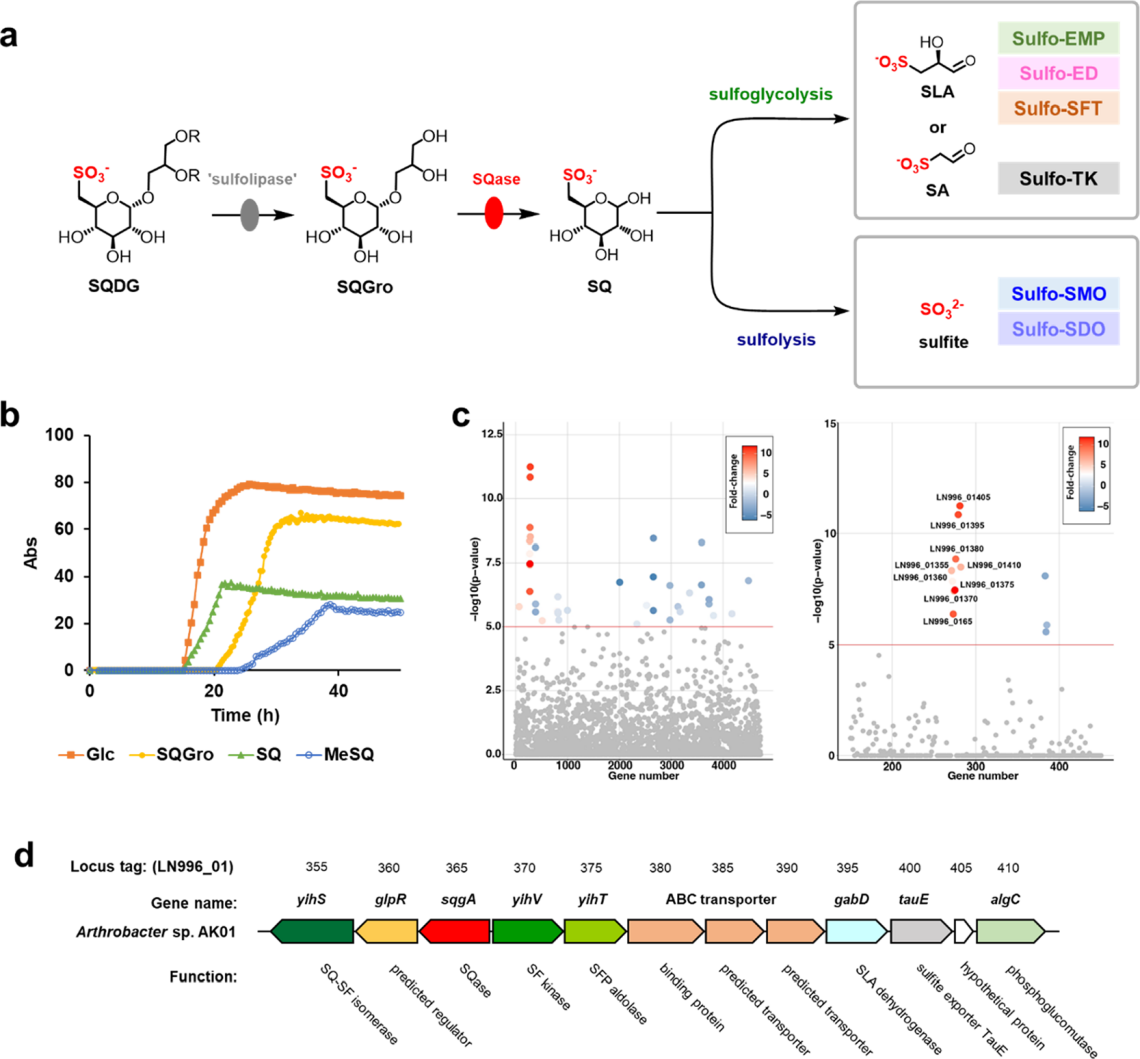
Identification of a new sulfoquinovose glycosidase, SqgA, in *Arthrobacter* sp. strain AK01**.** (a) Degradation of sulfoquinovosyl diacylglycerol (SQDG; R = acyl) to sulfoacetaldehyde (SA) or sulfolactaldehyde (SLA) by sulfoglycolysis (sulfoglycolytic Embden–Meyerhof–Parnas (sulfo-EMP); sulfoglycolytic Entner–Doudoroff (sulfo-ED); sulfoglycolytic sulfofructose transaldolase (sulfo-SFT); sulfoglycolytic transketolase (sulfo-TK) pathways) or sulfolysis to sulfite (sulfolytic SQ monooxygenase (sulfo-SMO); and sulfolytic SQ dioxygenase (sulfo-SDO) pathways). (b) Growth curve of *Arthrobacter* sp. AK01 grown on equimolar concentration (5 mM) of glucose, sulfoquinovose (SQ), sulfoquinovosyl glycerol (SQGro), or MeSQ. An independent replicate is shown in Figure S3. (c) Comparative proteomics of *Arthrobacter* sp. AK01 grown on glucose or SQ. (d) Gene cluster encoding the sulfo-EMP pathway for *Arthrobacter* sp. AK01.

The mineralization of SQ occurs through various sulfoglycolytic and sulfolytic pathways that enable utilization of its carbon and sulfur content (Figures 1a and S1, S2).^10−12^ Sulfoglycolytic pathways break the sugar chain to release C2 or C3 organosulfonates, which are substrates for secondary biomineralization pathways that catabolise these short-chain organosulfonates and release inorganic sulfite. Sulfolytic pathways cleave the C–S bond to release sulfite and provide glucose to fuel glycolysis. In all cases, these pathways require cleavage of the glycosidic bond present within sulfolipid or its delipidated form, sulfoquinovosyl glycerol (SQGro). Genes encoding sulfoquinovosidases (SQases) are diagnostic of SQ-utilizing gene clusters^13,14^ and are thus useful for discovery of new SQ degradation pathways,^15^ and for making functional inferences in (meta)genomic data sets.^8^

To date, only one class of SQase has been identified.^13,14^ It forms a subfamily of the carbohydrate active enzyme (CAZy) family of glycoside hydrolase 31 (GH31).^16^ Bioinformatic studies have shown that gene clusters encoding sulfoglycolytic and sulfolytic SQ degradation pathways often include genes encoding family GH31 SQases.^15^ However, some organisms harboring SQ degradation pathways lack GH31 SQases,^6,17^ raising questions as to how such organisms can utilize SQ glycosides. We recently reported two *Arthrobacter* spp. soil bacteria that contain a sulfoglycolytic Embden–Meyerhof–Parnas (sulfo-EMP) pathway but lack a candidate GH31 SQase, yet grow robustly on the SQ glycoside methyl α-sulfoquinovoside (SQMe) as sole carbon source.^17^ Here, we identify an alternative approach to sulfoquinovoside hydrolysis that is adopted by these organisms and discover a family of SQases belonging to a new glycoside hydrolase family (GH188). These new SQases operate through an oxidoreductive mechanism that results in net hydrolysis and involves a catalytic NAD^+^ cofactor. We show that these NAD^+^-dependent SQases adopt a complementary distribution to that of GH31 SQases and provide the missing SQase functionality in diverse sulfoglycolytic and sulfolytic gene clusters. Genes encoding these enzymes occur in bacteria, eukaryotes, and archaea and are especially abundant within marine bacteria.

## Results

To confirm the ability of *Arthrobacter* sp. strain AK01^17^ to grow on SQ glycosides, we examined growth on the naturally occurring glycoside, SQGro, which is formed by delipidation of SQDG (through action of nonspecific sulfolipases). *Arthrobacter* sp. strain AK01 exhibited robust growth and produced only sulfolactate in culture media, showing that glycerol and half of the SQ molecule were consumed, and thus that SQGro is cleaved to give SQ and glycerol (Figures 1b and S3). Growth curves for *Arthrobacter* sp. strain AK01 on 5 mM Glc or SQGro achieved peak optical density approximately twice that of 5 mM SQ or SQMe, suggesting that Glc and SQGro function as 6-carbon substrates, while SQ and SQMe function as 3-carbon substrates. To identify the molecular basis of sulfoglycolysis in this organism, we grew strain AK01 on SQ to remove complicating effects from coincident glycerol catabolism with SQGro. Cultures were independently grown on SQ or glucose, and comparative proteomics was conducted. Proteins displaying increased abundance belong to the previously identified sulfo-EMP pathway, including SQ-sulfofructose (SF) isomerase, SF kinase, SF-1-phosphate (SFP) aldolase and sulfolactaldehyde dehydrogenase (Figure 1c,d). Also identified was a protein (LN996_0165) that belongs to the short-chain dehydrogenase/reductase (SDR) superfamily, a large grouping of nicotinamide-cofactor dependent oxidoreductases with activity on diverse substrates.^18^ Preliminary analysis revealed that sequence-related homologues were associated with other *Arthrobacter* spp. that possessed syntenic sulfo-EMP gene clusters. Based on data as described later, we named this enzyme SQ glycosidase, SqgA.

**Figure 2:**
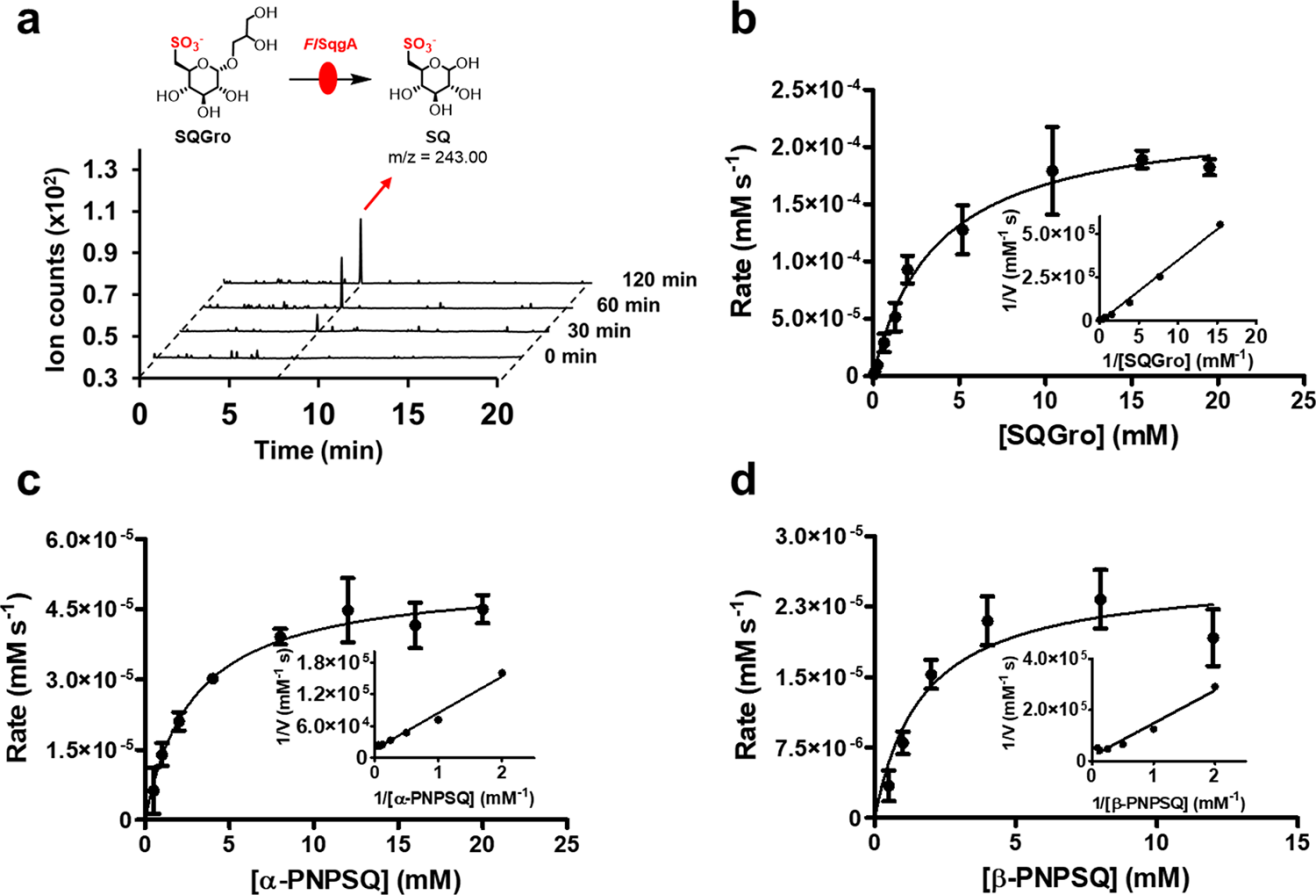
SqgA is a sulfoquinovosidase**.** (a) HPLC mass spectra (triple quadrupole, QqQ) chromatograms showing *Flavobacterium* sp. *Fl*SqgA catalyzed conversion of SQGro (total ion chromatogram MS^2^ of *m*/*z* = 317.08) to SQ (total ion chromatogram MS^2^ of *m*/*z* = 243.00) at time (*t*) = 0, 30, 60, and 120 min (b–d) Michaelis–Menten and Lineweaver–Burk plots for reaction rates measured for *Fl*SqgA catalyzed hydrolysis of SQGro, α-PNPSQ, and β-PNPSQ, respectively.

We synthesized several genes of homologues (Supplementary experimental) with codons harmonized for *E. coli* and screened their expression. *Fl*SqgA from *Flavobacterium* sp. strain K172 was selected for further study. Optimization of the reaction conditions revealed that incubation of *Fl*SqgA with DTT and NAD^+^ led to cleavage of SQGro to give SQ (Figure 2a). Experiments that involved the omission of DTT or NAD^+^ did not lead to the formation of SQ. Inclusion of Mn^2+^ led to an increase in product formation; however, kinetic analysis revealed no improvement in rate in the presence of Mn^2+^, and inclusion of EDTA caused a partial reduction of enzyme activity (Figure S4). Further analysis revealed that the small increase in product formation observed in the presence of Mn^2+^ was a result of improved enzyme stability; thus, Mn^2+^ was included in subsequent kinetic studies. These data show that *Fl*SqgA is a metal-independent NAD^+^-dependent SQase. Michaelis–Menten parameters for SQGro were measured by liquid chromatography–mass spectrometry using a triple-quadrupole mass spectrometer. This showed *k*_cat_ = 0.24 ± 0.02 s^–1^, *K*_M_ = 3.7 ± 0.9 mM, and *k*_cat_/*K*_M_ = 0.07 ± 0.02 mM^–1^ s^–1^ (Figure 2b). Another feature of NAD^+^-dependent glycosidases is the ability of many members to act on both α- and β-linked glycosides.^19^ Both 4-nitrophenyl α- and β-sulfoquinovosides (α-PNPSQ and β-PNPSQ) were substrates with the following Michaelis–Menten kinetic parameters: α-PNPSQ, *k*_cat_ = 0.025 ± 0.002 s^–1^, *K*_M_ = 2.9 ± 0.8 mM, and *k*_cat_/*K*_M_ = 0.012 ± 0.001 mM^–1^ s^–1^; β-PNPSQ, *k*_cat_ = 0.014 ± 0.002 s^–1^, *K*_M_ = 1.7 ± 0.6 mM, and *k*_cat_/*K*_M_ = 0.008 ± 0.001 mM^–1^ s^–1^ (Figure 2c,d). No activity was observed against 4-nitrophenyl α- and β-glucosides or 4-nitrophenyl β-glucuronic acid, showing that *Fl*SqgA is specific for SQ glycosides (Figure S4). The Michaelis activation constant for NAD^+^ at a constant α-PNPSQ concentration was *K*_A_ = 0.16 ± 0.02 mM (Figure S5). Two other orthologues, *Ar*SqgA from *Arthrobacter* sp. U41 and *Cr*SqgA from *Cryobacterium* sp. TMT2–4, also cleaved α-PNPSQ (Figure S6).

**Figure 3:**
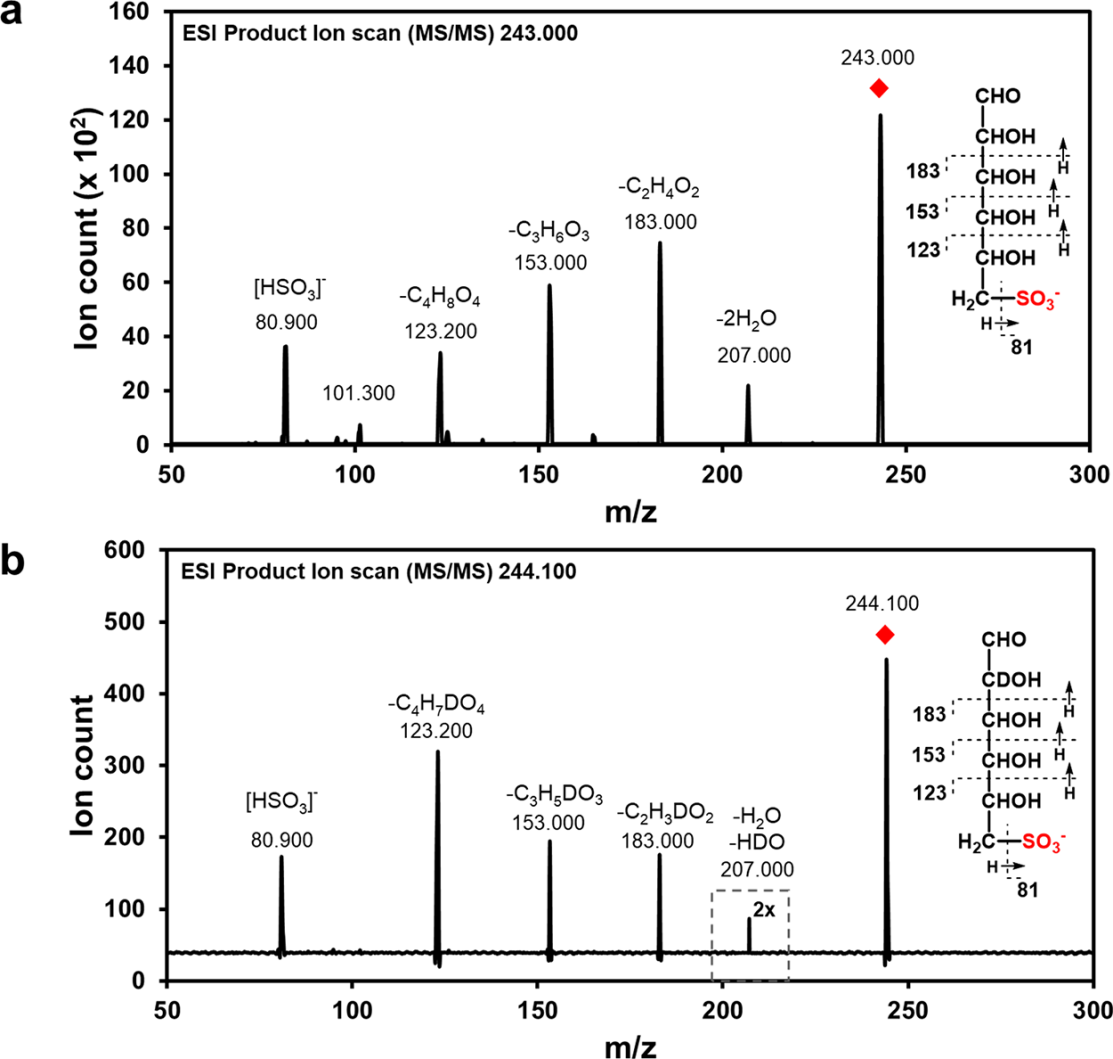
MS–MS fragmentation of deuterium-labeled SQ**.** (a) Fragment ions of [M-H]^−^ ions of SQ. (b) Fragment ions of [M-H]^−^ ions of deuterium-labeled SQ from the reaction mixture containing SQGro and *Fl*SqgA in D_2_O. No change in the *m*/*z* values of the fragments at nominal *m*/*z* 123, 153, and 183 shows that D is not attached to C3, C4, C5, or C6. The fragmentation of *m*/*z* 244 → 207 in both cases is proposed to arise from elimination of H_2_O/HOD across C2–C3 and H_2_O across C4–C5. Collectively, these data support incorporation of D at C2.

NAD^+^-dependent glycoside hydrolases have been described that belong to CAZy GH families 4, 109, 177, and 179.^19^ Among these, the best studied are those belonging to GH family 4, which are Mn^2+^-dependent and have been shown to operate through a multistep mechanism that involves oxidation at C3 to a ketone, elimination of the glycoside to give an α,β-unsaturated ketone, hydration and then reduction of the ketone to give the sugar hemiacetal.^20^ A diagnostic feature of this pathway is the incorporation of deuterium at C2 when the reaction is conducted in D_2_O, through hydration of the intermediate α,β-unsaturated ketone. A similar mechanism is invoked for enzymes of GH109, which like *Fl*SqgA studied here, are metal-independent.^21^ We therefore incubated *Fl*SqgA and the chromogenic glycoside 4-nitrophenyl α-sulfoquinovoside (α-PNPSQ) in D_2_O and analyzed the product by mass spectrometry. Upon exchange to H_2_O, the molecular ion ([M-H]^−^) increased by *m*/*z* of 1, consistent with incorporation of a nonexchangeable D atom. Fragmentation of the resulting [M-H]^−^ ion with collision-induced dissociation supports the incorporation of D at C2 (Figure 3).

**Figure 4:**
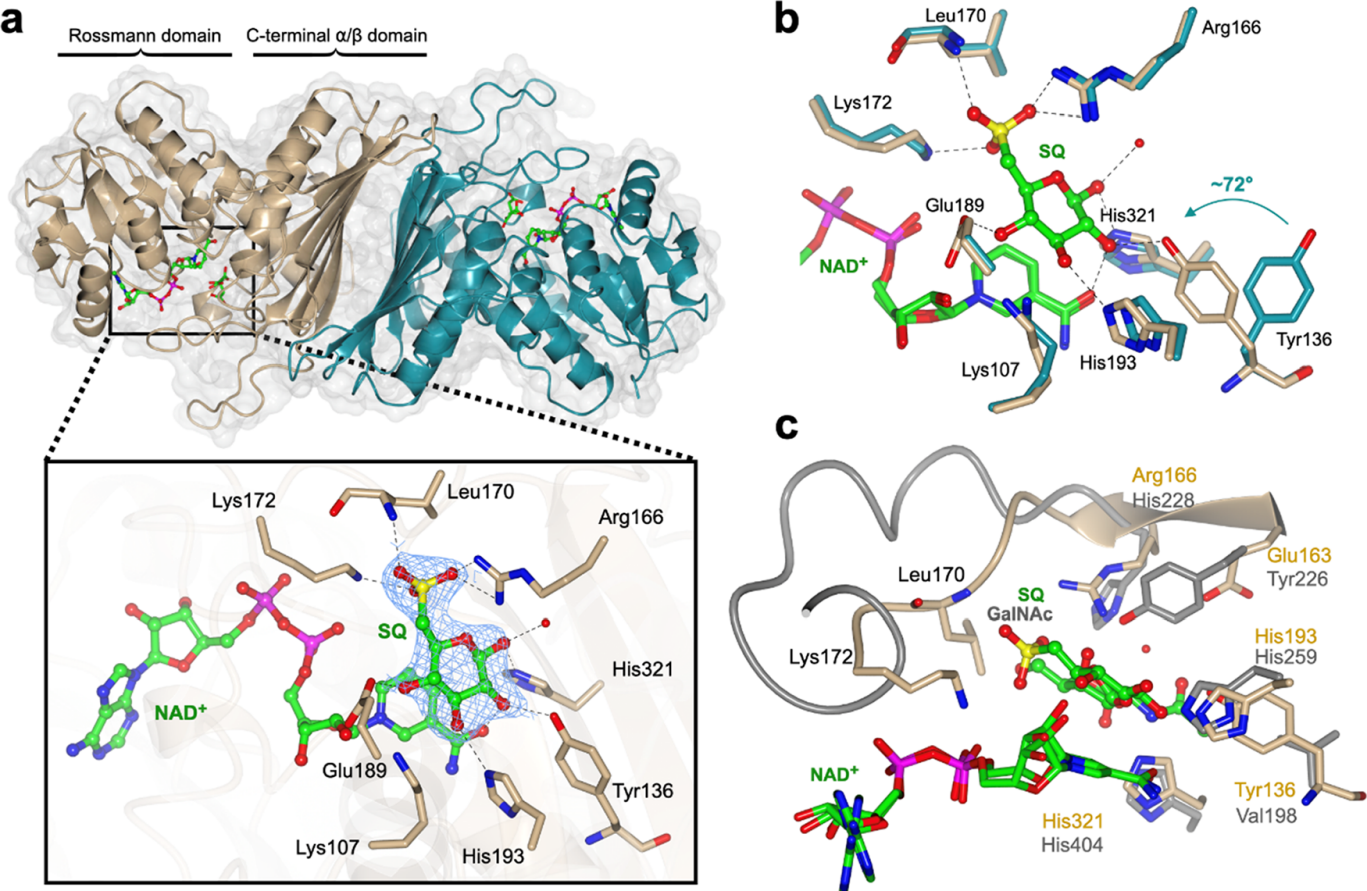
3D crystal structures of *Ar*SqgA**.** (a) 3D structure showing the quaternary structure (subunits shown in beige and dark cyan) and interactions of ligands, NAD^+^ and SQ, with the active site residues. Electron density in blue mesh corresponds to σA-weighted 2*F*_o_–*F*_c_ map contoured at 1σ (0.2783 electrons per Å^3^). (b) Overlay of *Ar*SqgA·NAD^+^ (in dark cyan) and *Ar*SqgA·NAD^+^·SQ (in beige) complexes show the rotation of Tyr136 upon binding SQ. (c) Superposition of *Ar*SqgA·NAD^+^·SQ (in beige) and *Am*GH109A·NAD^+^·GalNAc (in gray) complexes highlight conservation of catalytic residues and unique sulfonate binding residues in SqgA enzymes. Residues 164–172 (in beige) in the loop harboring sulfonate binding residues of *Ar*SqgA and corresponding longer loop region (residues 228–242 in gray) of *Am*GH109 are shown in ribbon format.

To gain insight into the molecular basis of catalysis, we determined the 3D structures of *Fl*SqgA and *Ar*SqgA using X-ray crystallography. Crystals of *Fl*SqgA and *Ar*SqgA were grown in the presence of NAD^+^ (or a ternary complex of *Ar*SqgA with citrate and NAD^+^) and diffracted to 2.35 and 2.65 (or 1.95) Å, respectively (Tables S1 and S2). The structures reveal homodimers, consistent with molar mass analysis using size-exclusion chromatography-multiangle laser light scattering in solution (Figures 4a and S7–S9). SqgA proteins dimerize through hydrophobic and polar interactions over a nine-stranded, flat β-sheet surface that buries 7468 Å^2^ at the dimerization interface, corresponding to 22% of the monomer surface. The SqgA proteins adopt a two-domain fold with an N-terminal dinucleotide binding Rossmann domain and a C-terminal α/β dimerization domain. Clear, contiguous density was seen in both structures within a cleft between the two domains that allowed modeling of a single molecule of NAD^+^ bound in an extended conformation in each monomer with its nicotinamide ring projecting into a polar pocket.

Soaking and cocrystallization experiments provided structures of the *Ar*SqgA·NAD^+^·SQ and *Fl*SqgA·NAD^+^·SQ complexes, which diffracted to 2.4 and 2.3 Å, respectively (Figures 4b and S10). Both ternary complexes were essentially identical, with respect to the active site interactions. In the *Ar*SqgA·NAD^+^·SQ complex, SQ is situated above the nicotinamide ring, with C3 of SQ 3.4 Å away from C4 of the nicotinamide ring, at an appropriate distance for hydride transfer and oxidation of C3 of an SQ glycoside. A range of specific interactions occur with the hydroxyl groups of SQ: C1–OH at 2.5 Å from His321 and a water molecule; C2-OH with Tyr136 (2.6 Å); C3–OH with His193 (2.7 Å) and Lys107 (2.6 Å); and C4–OH with Glu189 (2.6 Å; Figure 4b). The characteristic 6-sulfonate group of SQ engages in a triad of interactions in the *Ar*SqgA·NAD^+^·SQ: one oxygen H-bonds to Arg166 (2.6 Å), a second to Lys172 (2.9 Å), and a third to the backbone amide of Leu170 (2.8 Å). Superposition of *Ar*SqgA·NAD^+^ binary and *Ar*SqgA·NAD^+^·SQ ternary complexes (backbone RMSD of 0.5 Å over 364 residues) revealed no major structural changes upon binding SQ. The active site was also largely unchanged with the notable exception of the Tyr136 residue in some of the individual protomer chains, which rotated 72° about the Cα–Cβ bond to engage with C2–OH of SQ in the *Ar*SqgA·NAD^+^·SQ complex. All of these residues are conserved across the studied SqgA homologues (Figure S11).

Structural comparison of SqgA enzymes using the DALI server matched various oxidoreductases with high *Z*-score of >33, despite very low sequence similarity. These include an inositol-2-dehydrogenase (IDH) (PDB: 4MIE), backbone RMSD of 1.87 Å over 279 aligned residues and 19% sequence identity; a glucose-fructose/IDH/MocA-like oxidoreductase (PDB: 1ZH8) with backbone RMSD 2.1 Å over 313 residues and 24% sequence identity; and intriguingly, an NAD^+^-dependent *N*-acetylgalactosaminidase, *Am*GH109A, belonging to family GH109 (PDB ID: 6T2B with backbone RMSD 2.22 over 293 residues, 15% sequence identity). As noted earlier, GH109 glycosidases are NAD^+^-dependent but metal-independent enzymes, acting through an analogous oxidoreductive mechanism. In the case of *Am*GH109A, His404 is proposed to act as base/acid in the oxidation/reduction of C3, Tyr226 as general base/acid for proton transfer at C2, and His259 as general acid/base for cleavage of the glycosidic linkage and addition of water.^22^ Superposition of the *Ar*SqgA·NAD^+^·SQ and *Am*GH109A·NAD^+^·GalNAc complexes shows that His259 (His193 in *Ar*SqgA) and His404 (His321 in *Ar*SqgA) are structurally conserved (Figure 4c). However, a difference occurs in the active site with respect to the location of Tyr226 of *Am*GH109A, where Glu163 occupies the equivalent position in *Ar*SqgA; and Tyr136 hydrogen-bonds to C2–OH of SQ in our structure, implicating it in catalysis. Comparison of the superimposed structures of *Ar*SqgA·NAD^+^·SQ with those of *Am*GH109A·NAD^+^·GalNAc and the Mn^2+^-dependent family GH4 member BglT·NAD^+^·G6P (from *Thermotoga maritima*, PDB: 1UP6) revealed conserved N-terminal domain folds, and cofactor and substrate binding sites, but differences in the C-terminal domain and active site interactions (Figures S12 and S13). Notably, BglT contains a well-defined Mn^2+^ coordination site that includes conserved cysteine (Cys162) and histidine (His192) residues, whereas both metal-independent enzymes lack a metal binding site at the equivalent position.

**Figure 5:**
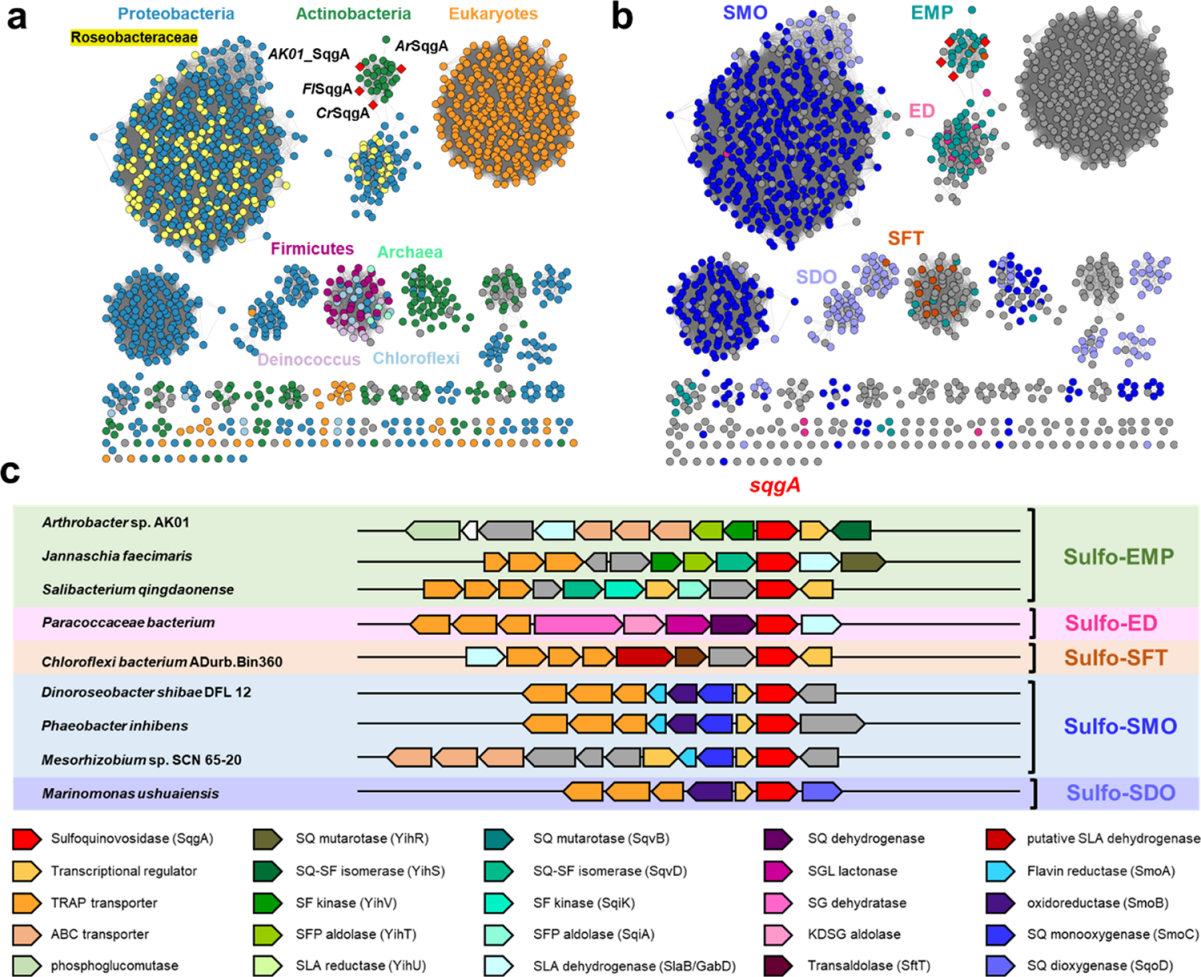
Genes encoding SqgA homologues occur in diverse SQ degrading gene clusters and are distributed across bacteria and eukaryotes**.** (a) SSNs of SqgA homologues at alignment score 133 (i.e., >53% identity) colored based on taxonomy of organisms harboring *sqgA* gene. (b) SSNs colored based on genetic context of SQase gene within proposed sulfo-EMP, sulfo-ED, sulfo-SFT, sulfo-SMO, and sulfo-SDO gene clusters. (c) Gene clusters encoding representative sulfoglycolysis (sulfo-EMP/ED/SFT) and SQ sulfolytic (sulfo-SMO/SDO) pathways. Protein accession codes are *Arthrobacter* sp. AK01 (MCD4849452.1), *Jannaschia faecimaris* (A0A1H3MHE8), *Salibacterium qingdaonense* (A0A1I4NIZ9), *Paracoccaceae bacterium* (A0A7Z8PBZ1), *Chloroflexi bacterium* ADurb.Bin360 (A0A1 V5Q144), *Dinoroseobacter shibae* DFL12 (A8LIZ7), *Sphinomonas* sp. Leaf17, (A0A0Q4FI28), *Mesorhizobium* sp. SCN 65–20 (A0A1D2SWD9), *Phaeobacter inhibens* (A0A135IMB2), and *Marinomonas ushuaiensis* (X7E4N4).

These NAD^+^-dependent SQases serve as prototypes for a new GH family. We used the C-terminal domain of AK01 (lacking the NAD^+^-binding domain) as a query to search for related sequences on the phmmer database that were used to build a hidden Markov model (HMM). Iterative searches and rebuilding of the HMM were conducted until a stable set of sequences was obtained containing approximately 1600 retrieved sequences (Figure S14). The retrieved sequences constitute a new GH family (GH188), which was studied further using sequence similarity network (SSN) analysis.^23^ We used analysis of network centrality^24^ to guide the selection of an appropriate alignment score for the SSN (Figures S15 and S16). At an alignment score of 133, this SSN breaks into clusters that align closely with taxonomy and showcases that these NAD^+^-dependent SQases are found in diverse bacteria, algae, plants, and a small number of archaea (Figure 5a). Eukaryotic members are present within agriculturally significant plants including *Oryza sativa*, *Zea mays*, *Hordeum vulgare*, *Theobroma cacao*, *Nicotinia tabacam*, and the red alga *Gracilariopsis chorda*. Bacterial representatives include *Faecalicatena contorta*, which has been isolated from human feces.^25^ We used the bacterial representatives to retrieve the genome neighborhood diagrams (open reading frame ± 10 around *sqgA*). Representative genome neighborhood diagrams reveal SqgA encoding genes are associated with sulfo-EMP, sulfo-ED, sulfo-SFT, sulfo-SMO, and sulfo-SDO pathways (Figures 5b,c, Table S3). These clusters lack a family GH31 SQase, and thus, the GH188 member will provide these organisms with a capacity to utilize SQ glycosides through these pathways. A significant observation is the near-universal occurrence of GH188 SQases in the sulfo-SMO gene clusters within marine *Roseobacter* clade bacteria (Figure S17). These organisms have previously been reported to lack GH31 SQases and were proposed to be restricted to growth only on the free sugar, SQ.^6^

## Discussion

The ubiquity of SQ within photosynthetic tissues means that it is a major species in the biosulfur cycle. Its significance as a nutrient is highlighted by the evolution of a variety of sulfoglycolytic and sulfolytic pathways for SQ catabolism. Organisms that seek to utilize SQ are faced with the challenge of cleaving it from its glycoconjugates, which are the major forms present in the natural world. Previously, a single class of SQases were identified that belongs to CAZy family GH31.^13,14^ However, experimental work and bioinformatic analysis have identified many organisms that are capable of growth on SQ-glycosides and/or which lack a gene encoding a candidate GH31 SQase. Here, we discover a new family of NAD^+^-dependent SQases with a widespread distribution that is complementary to classical GH family 31 SQases, and which provides the missing enzymatic capability.

**Figure 6:**
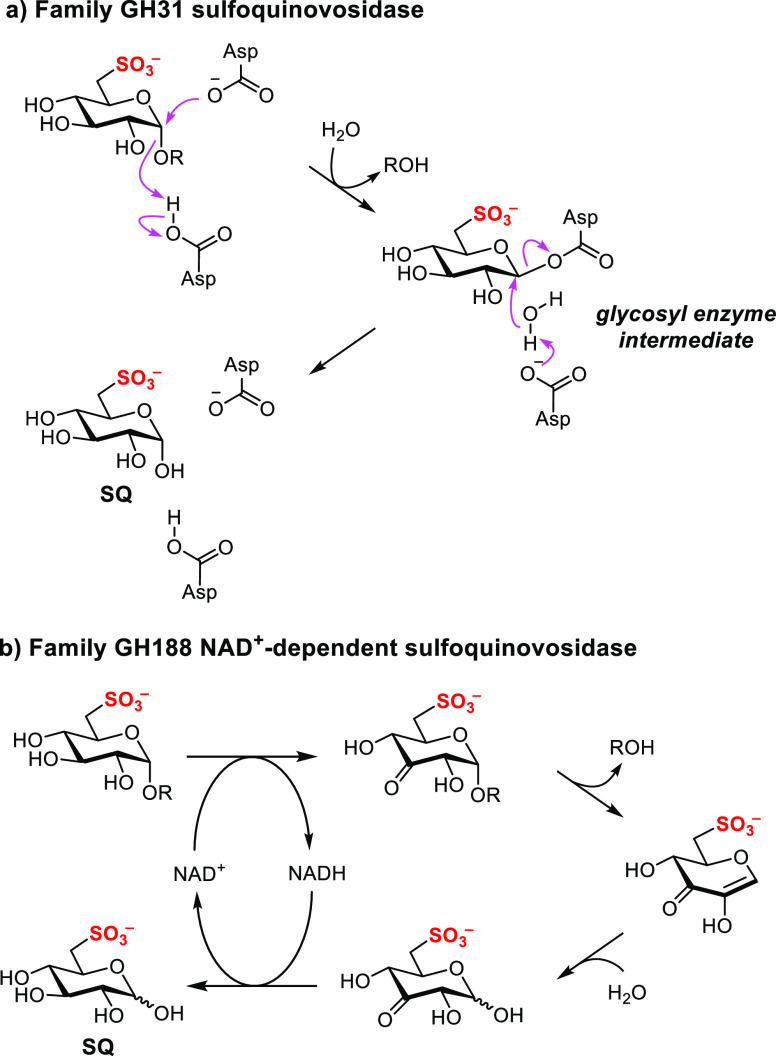
Proposed mechanisms for (a) family GH31 and (b) family GH188 NAD^+^-dependent sulfoquinovosidases.

The new family of NAD^+^-dependent SQases described here possesses the ability to cleave α- and β-linked SQ glycosides, in contrast to family GH31 SQases, which are specific for α-SQ glycosides. The ability to cleave α- and β-linked glycosides is a consequence of an oxidoreductive mechanism (with a rate-limiting transition state that does not involve C1–O cleavage)^20^ that is distinct from that used by family GH31 glycosidases (involving C1–O cleavage at the rate-limiting transition state) (Figure 6).^26^ Although not investigated here, it is tempting to suggest that these anomer nonspecific SQases could allow the release of SQ from sulfolipid and related species (α-linked) and SQ-containing N-glycans (β-linked).^3^

## Conclusions

Our work highlights that the distribution of NAD^+^-dependent SQases is complementary to that of classical GH31 SQases. We discover examples of NAD^+^-dependent SQase encoding genes within gene clusters of almost all known SQ degrading pathways, suggesting their importance, along with GH31 SQases, as gateway enzymes for SQ catabolism pathways. Of significance is the occurrence of NAD^+^-dependent SQases within *Roseobacter* clade bacteria. It has recently been argued that because *Roseobacter* clade bacteria lack a classical GH31 SQase, they cannot utilize SQ glycosides, and only utilize SQ, a very minor species within the overall budget of natural SQ-related molecules.^6^ In fact, essentially all *Roseobacter* clade bacteria investigated encode family GH188 SQases, highlighting that this new family of NAD^+^-dependent SQases contributes to SQ utilization within the marine environment. Collectively, the two classes of SQases that are now known provide a powerful bioinformatic signature for the identification of SQ degradation pathways and will support the survey of SQ degradation pathways across (meta)genomic data sets to provide a deeper understanding of the biogeochemical sulfur cycle.

## Data Availability

Atomic coordinate files and structure factors have been deposited in the Protein DataBank (PDB) with accession codes 8QC8 (*Fl*SqgA·NAD), 8QC2 (*Fl*SqgA·NAD·SQ), 8QC3 (*Ar*SqgA·NAD), 8QC6 (*Ar*SqgA·NAD·SQ) and 8QC5 (*Ar*SqgA·NAD·citrate). Data collection and refinement statistics are presented in Supplementary Tables S1 and S2. PFAM codes for neighborhood genes from different sulfoglycolytic and sulfolytic pathways are presented in Supplementary Table S3. A list of organisms containing homologous *sqgA* genes used for bioinformatics analysis is provided in Supporting Information file 2. Proteomics data has been deposited to the PRIDE partner repository with the data set identifier: PXD043482. The family classification for NAD^+^-dependent SQases is available at www.cazy.org.

## Supporting Information Available

Detailed experimental procedures, supplementary Figures and Tables: Figures S1–S17, Tables S1–S3 (PDF)
Supplementary Data. Accession codes and organism details for GH188 family members (XLSX)
