# Supplementary material for: Widespread Family of NAD+-Dependent Sulfoquinovosidases at the Gateway to Sulfoquinovose Catabolism
Source: J Am Chem Soc. 2023 Dec 15;145(51):28216–23. doi: 10.1021/jacs.3c11126 (PMC10755693; doi:10.1021/jacs.3c11126)
Supplement: Supplementary file 1 — ja3c11126_si_001.pdf [file ja3c11126_si_001.pdf]

SUPPLEMENTARY FIGURES AND TABLES

A widespread family of NAD$^+$-dependent sulfoquinovosidases at the gateway to sulfoquinovose catabolism

Arashdeep Kaur,[1,2] Isabelle B. Pickles,[3] Mahima Sharma,[3] Niccolay Madiedo Soler,[4,5] Nichollas Scott,[4] Sacha Pidot,[4] Ethan D. Goddard-Borger,[4,5]* Gideon J. Davies,[3]* Spencer J. Williams[1]*

[1] School of Chemistry, University of Melbourne, Parkville, Victoria 3010, Australia

[2] Bio21 Molecular Science and Biotechnology Institute, University of Melbourne, Parkville, Victoria 3010, Australia

[3] York Structural Biology Laboratory, Department of Chemistry, University of York, York YO10 5DD, United Kingdom

[4] ACRF Chemical Biology Division, The Walter and Eliza Hall Institute of Medical Research, Parkville, Victoria 3010, Australia

[5] Department of Medical Biology, University of Melbourne, Parkville, Victoria 3010, Australia

[6] Department of Microbiology and Immunology, University of Melbourne, at the Peter Doherty Institute for Infection and Immunity, Victoria 3000, Australia

Table of Contents

Supplementary Figures S1-S17	S2-S17
Supplementary Tables S1-S3	S18-S20
Experimental Methods	S21-S30
Supporting References	S30-S31

Supplementary Figures

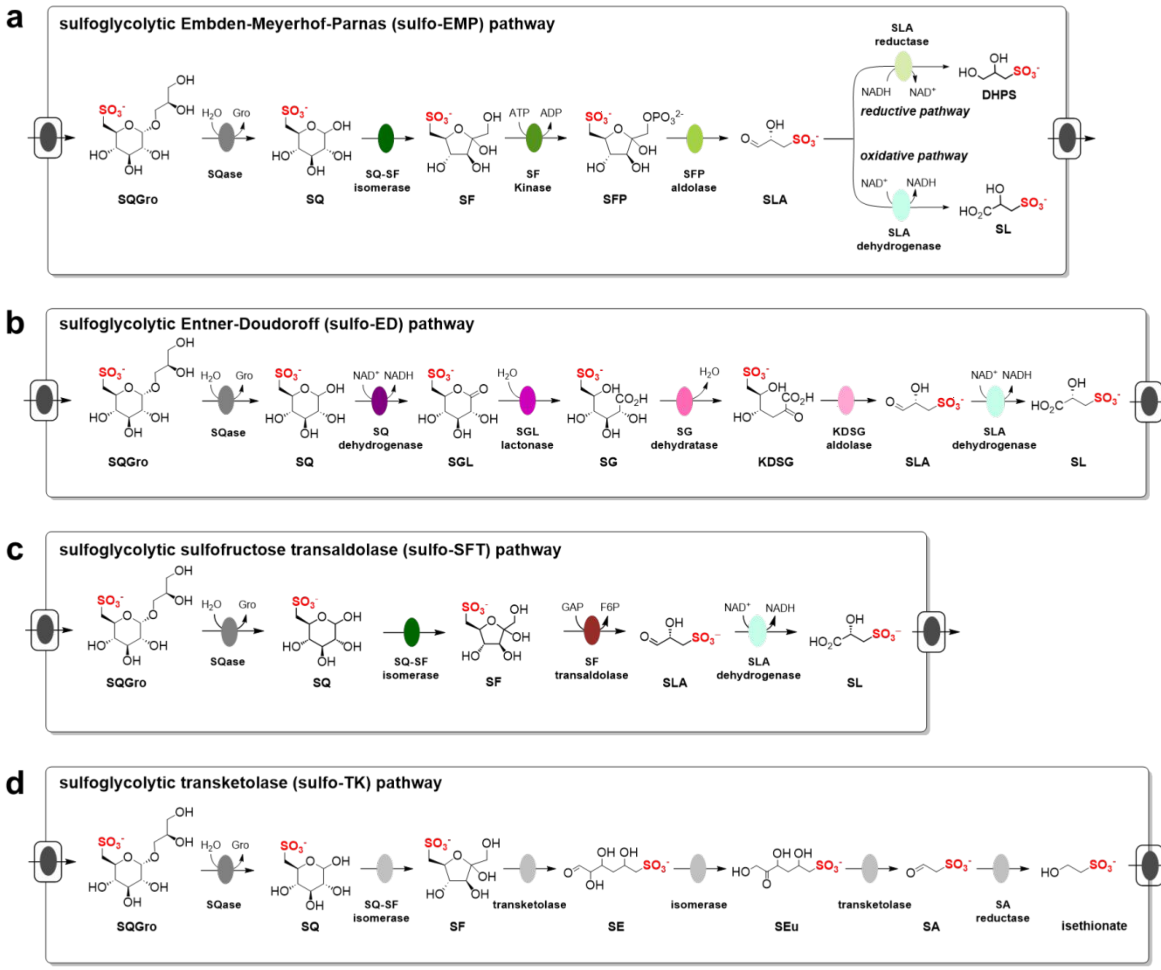

Figure S1. Sulfoglycolytic pathways: a) sulfoglycolytic Embden-Meyerhof-Parnas (sulfo-EMP) pathway, b) sulfoglycolytic Enter-Doudoroff (sulfo-ED) pathway, c) sulfoglycolytic sulfofructose transaldolase (sulfo-SFT) pathway.

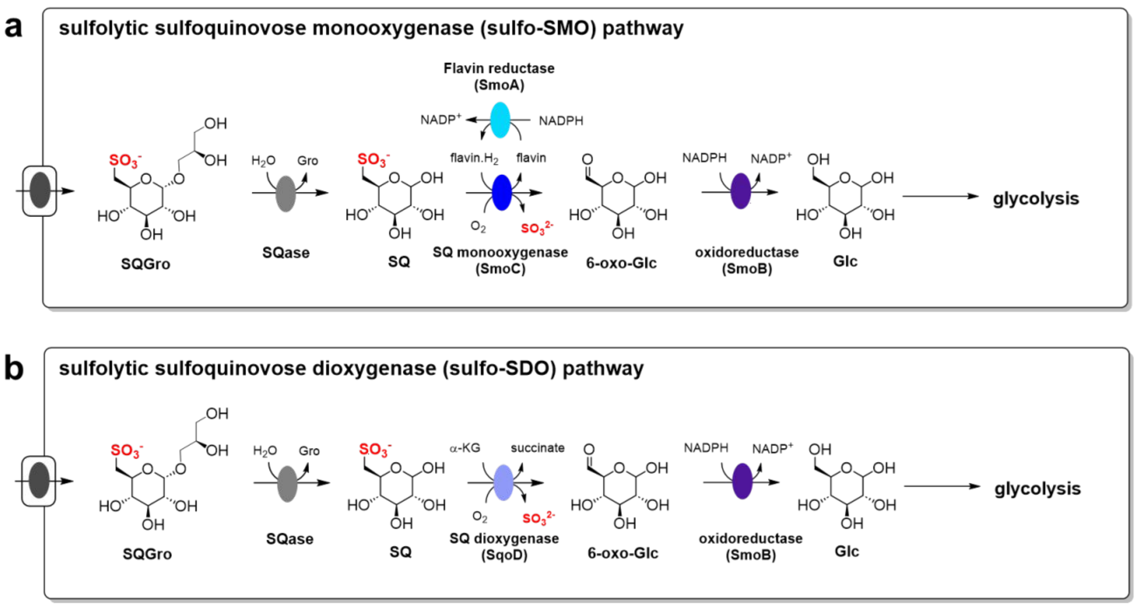

Figure S2. Sulfolytic pathways: a) sulfolytic sulfoquinovose monooxygenase (sulfo-SMO) pathway, b) sulfolytic sulfoquinovose dioxygenase (sulfo-SDO) pathway.

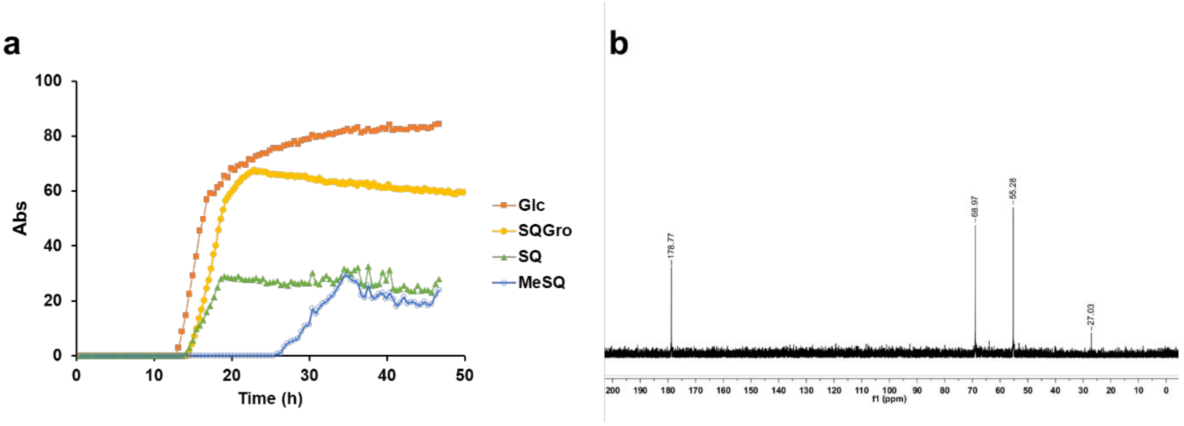

Figure S3. a) Growth curves of *Arthrobacter* sp. strain AK01 grown on minimal salts containing equimolar concentrations (5 mM) concentrations of SQGro, MeSQ, glucose or SQ. b) ^{13}C NMR spectra of spent culture of *Arthrobacter* sp AK01 grown on SQGro (5 mM).

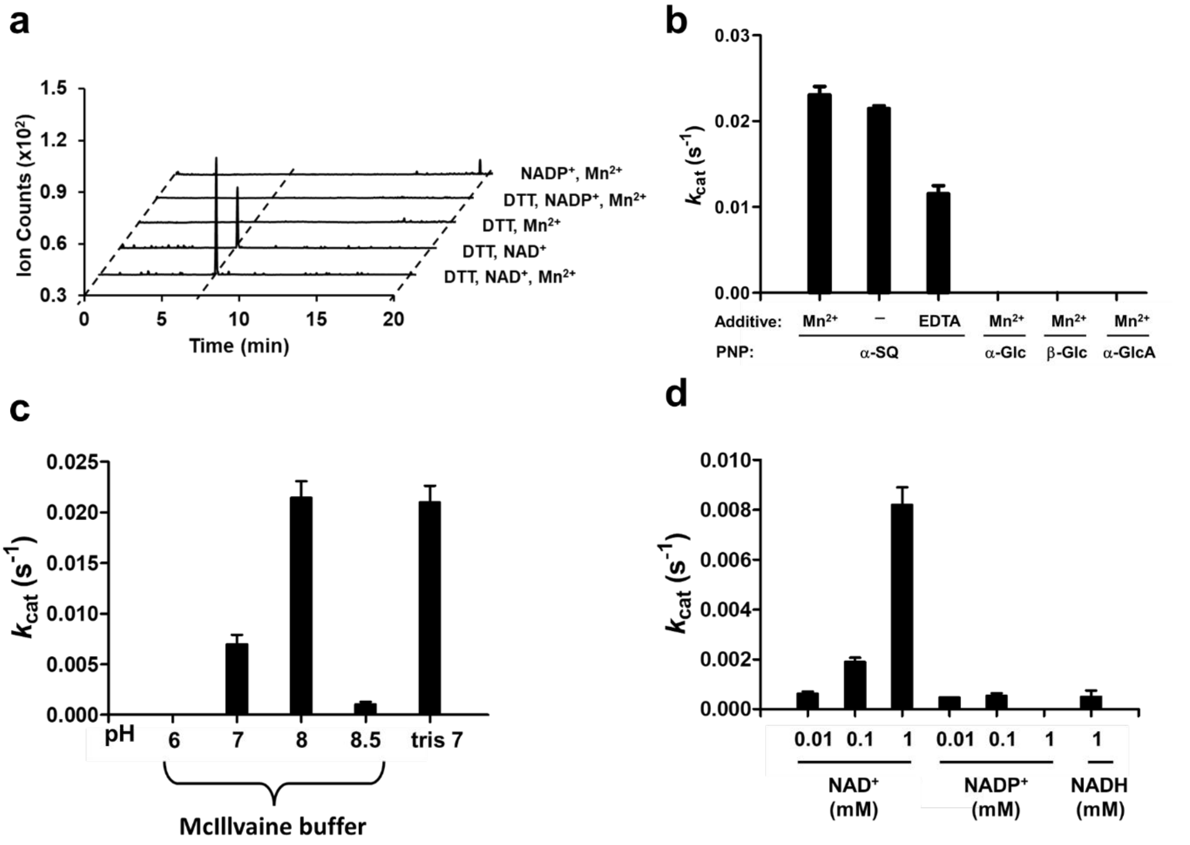

Figure S4. Optimization of reaction conditions for *Fl*SqgA and exploration of specificity. a) HPLC mass spectrometry (triple quadrupole, QqQ) chromatograms showing enzyme is active in the presence of NAD^+, DTT and optionally, Mn^{2+}. b) Enzyme activity assessed against various PNP glycosides, in the presence or absence of Mn^{2+}, and when treated with EDTA. c) pH profile for reaction of 10 mM α-PNPSQ with 1.86 μM of *Fl*SqgA. d) Enzyme activity in presence of NAD^+, $NADP^+$, or NADH.

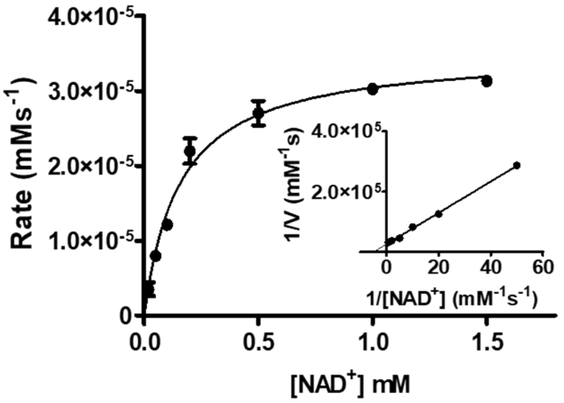

Parameter	Values
V_{max} (mM s^{-1})	(3.5 ± 0.1) × 10^{-5}
K_A (mM)	0.16 ± 0.02

Figure S5. Michaelis-Menten activation plot for *Fl*SqgA. Rates were measured using α-PNPSQ with a UV/Vis spectrometer.

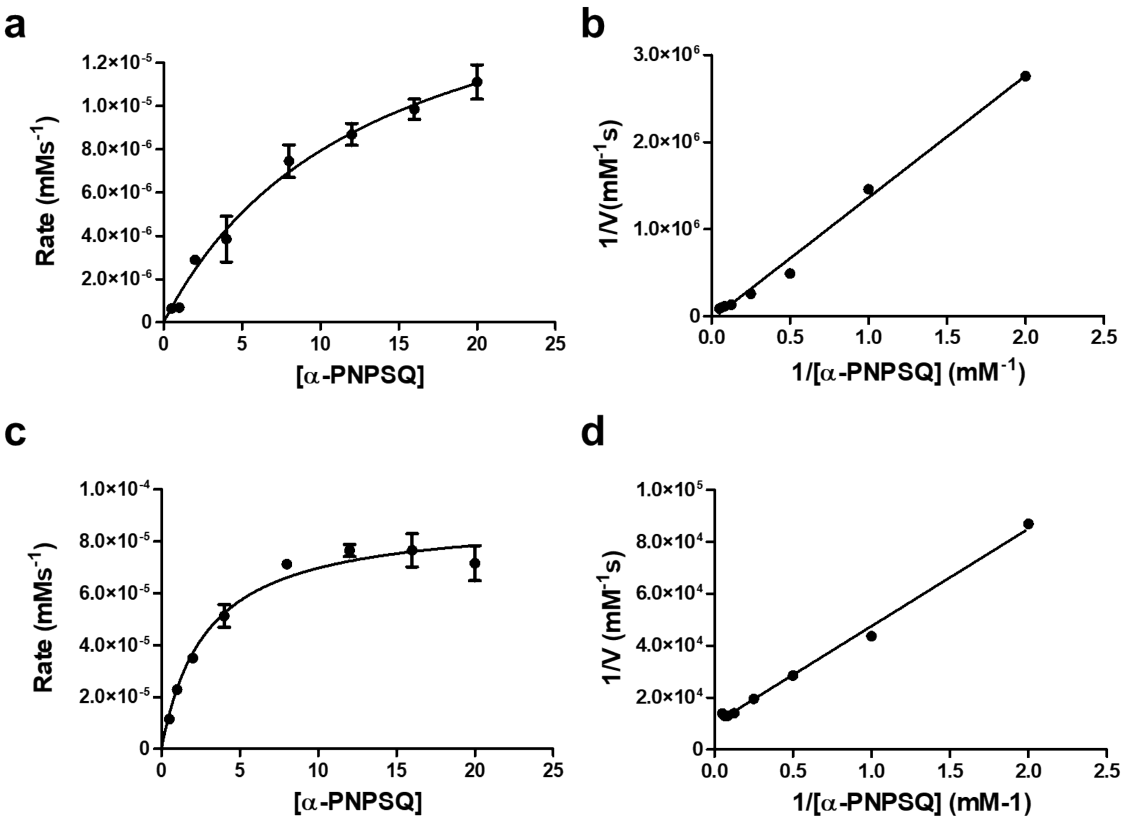

Figure S6. Kinetic analysis of *Ar*SqgA and *Cr*SqgA. a) and b) Michaelis-Menten and Lineweaver-Burk plots for reaction rates measured for *Ar*SqgA using α-PNPSQ as a substrate. c) and d) Michaelis-Menten and Lineweaver-Burk plots for reaction rates measured for *Cr*SqgA using α-PNPSQ as a substrate.

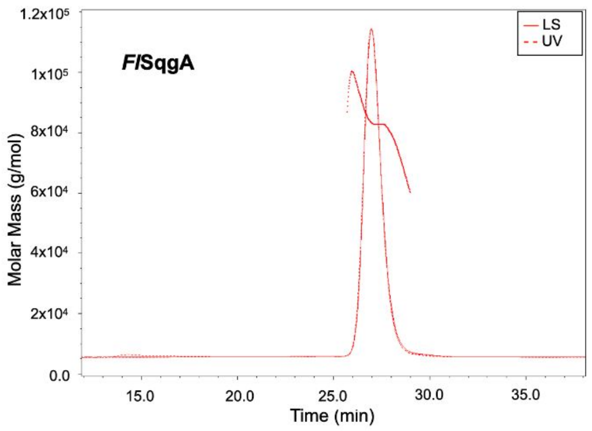

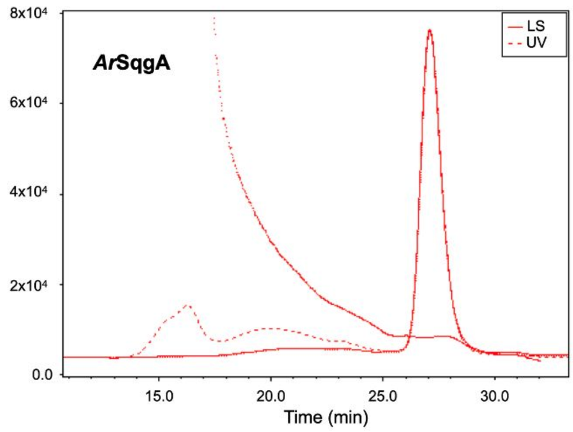

	**from**	**to (min)**	**amount (µg)**	**MW (kDa)**
***Fl*SqgA** (major peak)	25.7	29.0	285	83.6
***Ar*SqgA** (major peak)	25.9	29.0	63	83

Figure S7. Oligomeric assembly of SqgA enzymes in solution. Size exclusion chromatography-multiangle laser light scattering analysis for *Fl*SqgA (left) and *Ar*SqgA (right). The estimated molecular weight from both spectra is equivalent to a homodimer of each protein is shown in the table. Small amounts of higher order oligomers are also observed in the *Ar*SqgA sample perhaps due to four free, exposed cysteine residues.

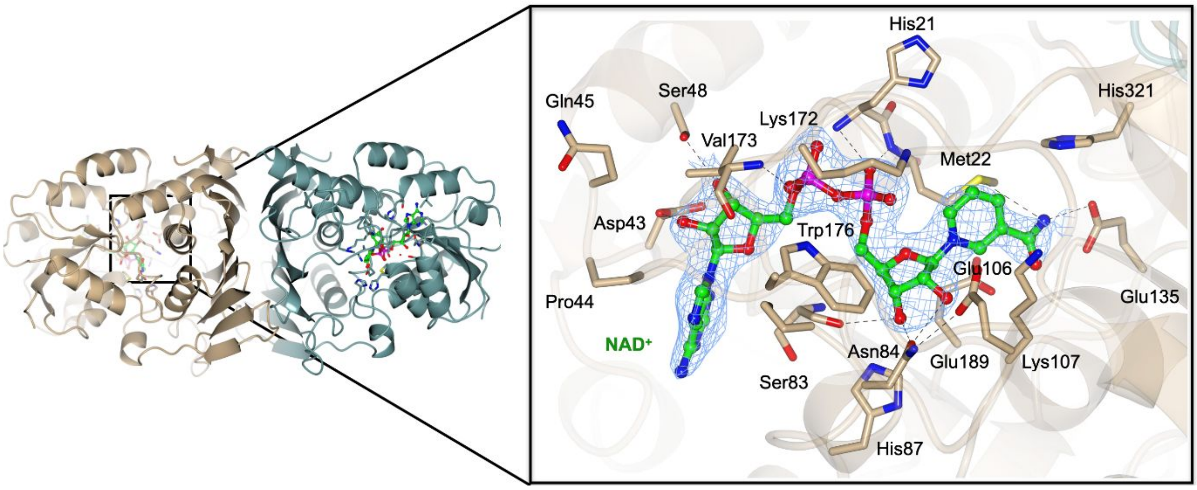

Figure S8. Crystal 3D structure of *Ar*SqgA•NAD$^+$ complex. Crystal structure showing the dimer pair shown in beige and dark cyan. The interactions with the active site residues within 4 Å vicinity of NAD$^+$ are shown on the left. Electron density in blue mesh corresponds to σ_A-weighted 2Fo − Fc map contoured at 1σ.

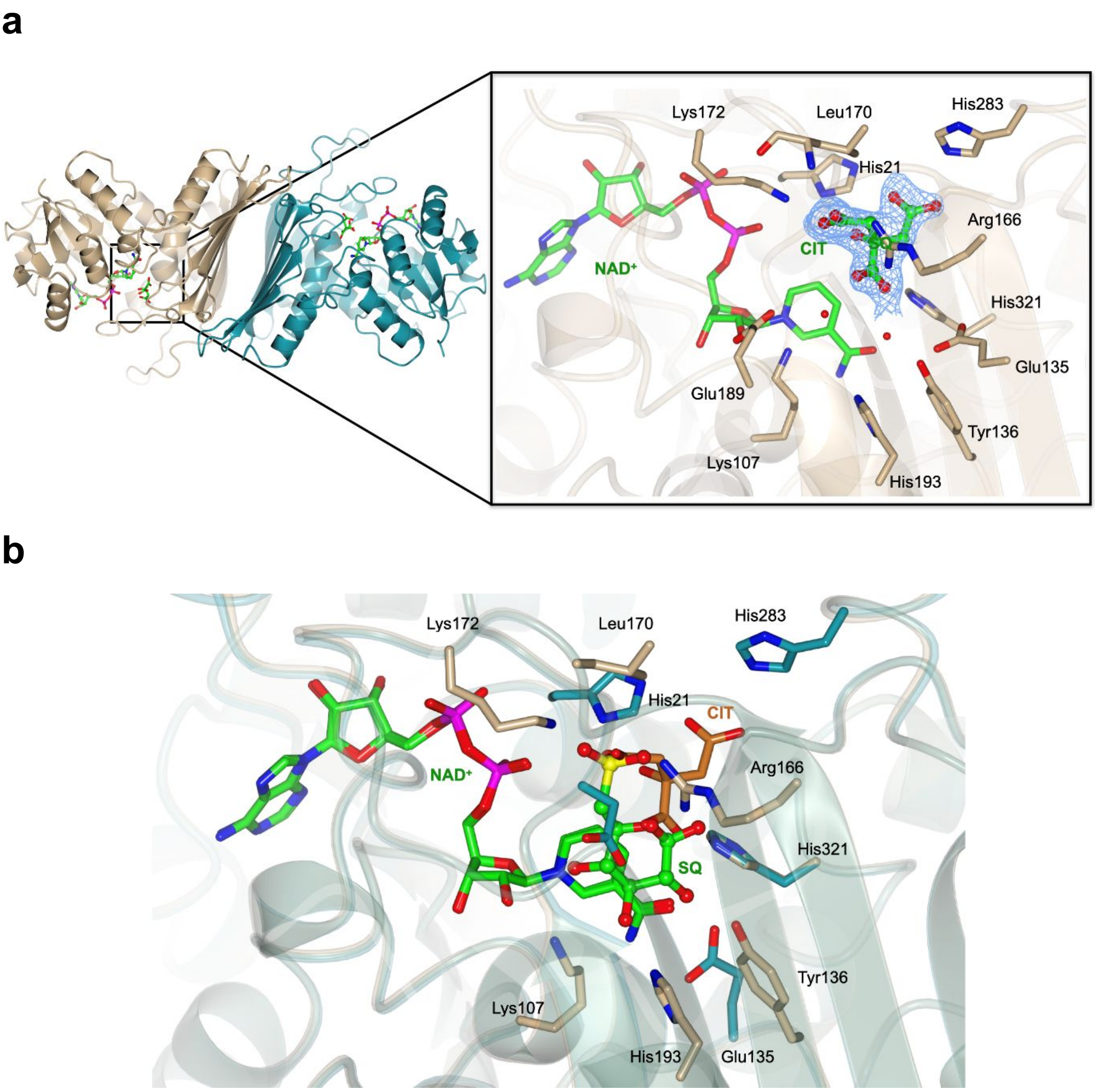

Figure S9. 3D crystal structure of *Ar*SqgA•NAD$^+$•citrate complex. a) 3D structure showing the physiological dimer pair and interactions of ligands, NAD$^+$ and citrate, with the active site residues. Citrate trianion (picked up from crystal growth conditions) H-bonds to Lys185, Arg179 and backbone amide of Leu183 through one β-carboxylate and the other β-carboxylate binds His34 and His296. The α-carboxylate makes hydrogen bonding interactions with His334 and two water molecules. Electron density in blue mesh corresponds to σ_A-weighted 2Fo − Fc map contoured at 1σ. b) Overlay of *Ar*SqgA•NAD$^+$•SQ (in beige) and *Ar*SqgA•NAD$^+$•citrate (in dark cyan) complexes show relative location of SQ and citrate trianion binding sites. Citrate molecule is shown in coral.

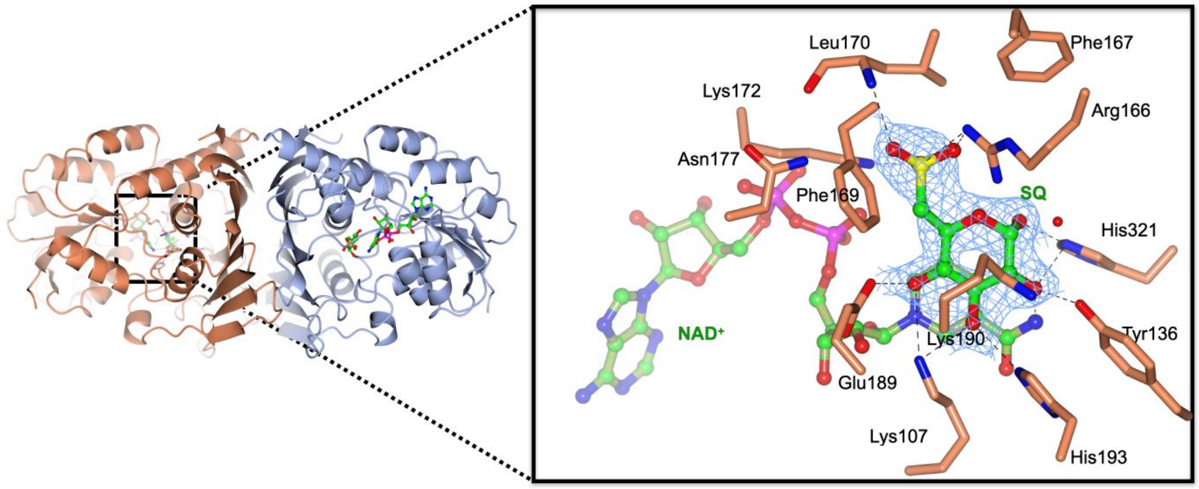

Figure S10. Crystal 3D structure of *Fl*SqgA•NAD⁺•SQ. 3D structure showing the physiological dimer pair and interactions of ligands, NAD⁺ and SQ, with the active site residues shown within 4 Å vicinity of SQ. Electron density in blue mesh corresponds to σ_A-weighted 2Fo − Fc map contoured at 1σ.

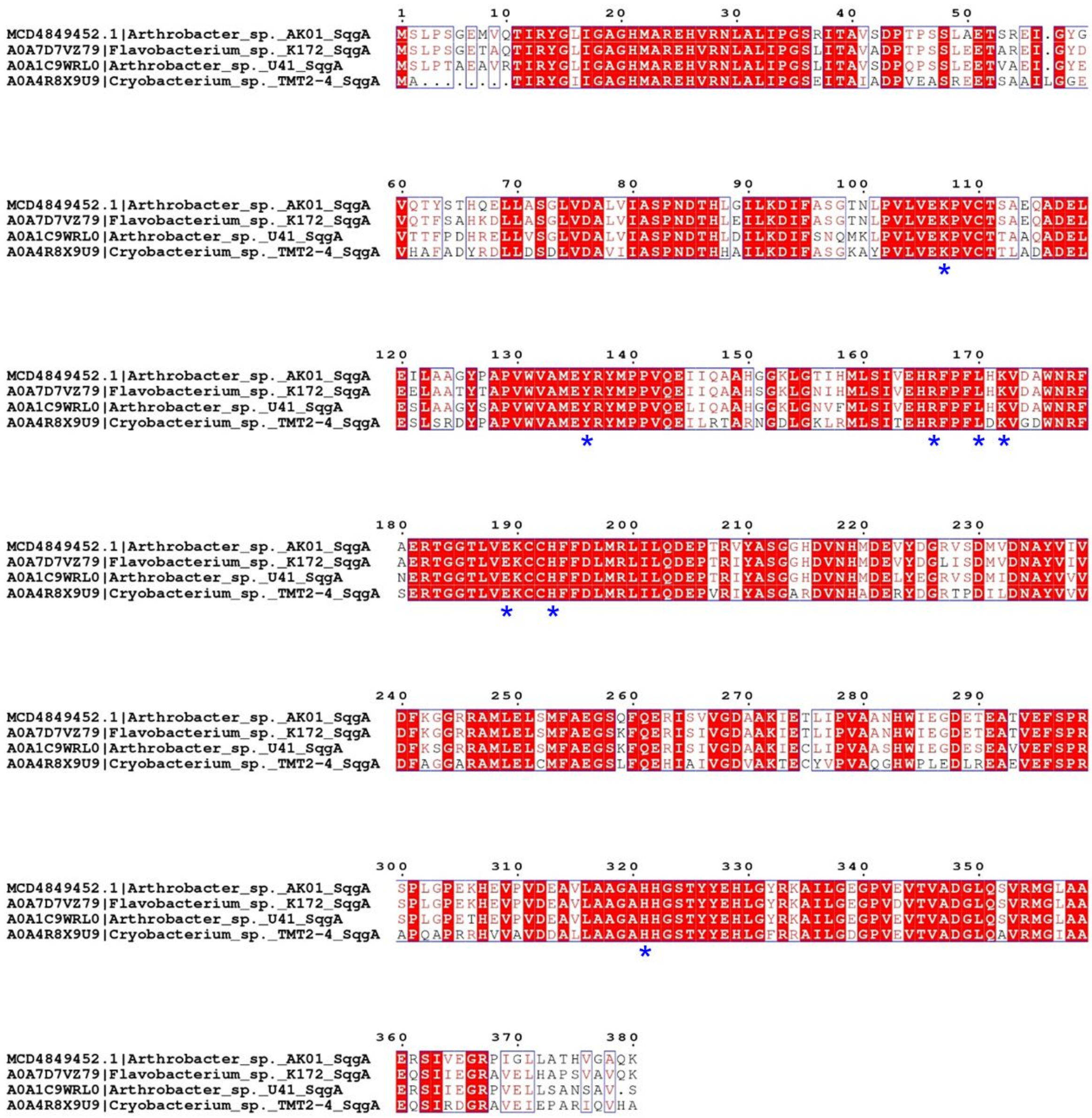

Figure S11. Multiple sequence alignment of SqgA from *Arthrobacter* sp. strain AK01, *Flavobacterium* sp. strain K172, *Arthrobacter* sp. U41 and *Cryobacterium* sp. TMT2-4**.**

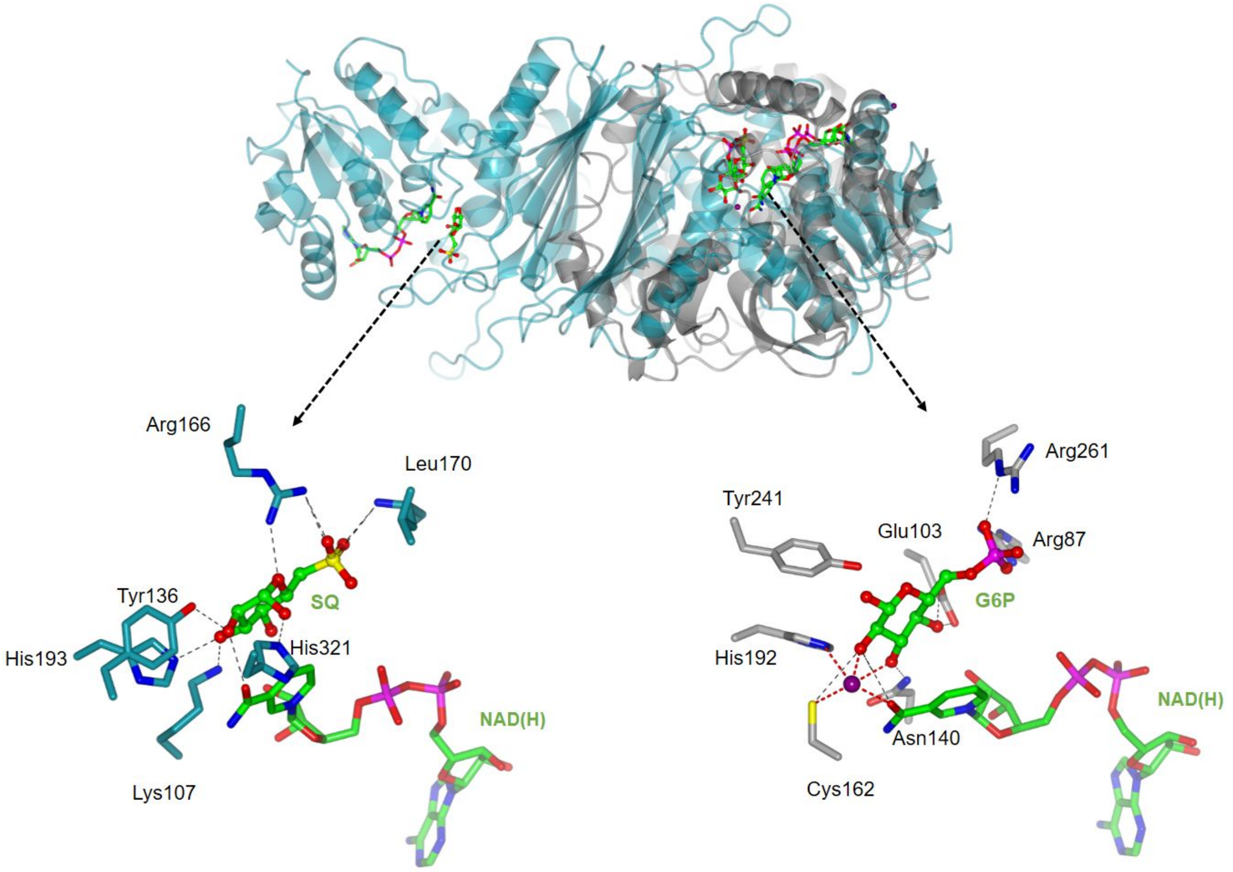

Figure S12. Superposition of structures of *Ar*SqgA•NAD$^+$•SQ (in dark cyan) with Mn^{2+}/NAD$^+$-dependent family GH4 BglT•NAD$^+$•G6P (PDB:1UP6 in grey). Overlay reveals similar N-terminal domain and location of cofactor and substrate binding sites, and highlights differences in the C-terminal domain and active site interactions between the two families. For clarity, only BglT monomer is superposed on *Ar*SqgA dimer (RMS deviation of 3.51 Å over 156 residues). For BglT, an Mn^{2+} binding site (shown in dark magenta) is observed, with metal interactions with glucose-6-phosphate C2-OH (2.1 Å) and C3-OH (2.5 Å), nicotinamide of NAD$^+$ cofactor, and the conserved active site residues Cys162 and His192 (at 2.4 Å and 2.5 Å, respectively). For *Ar*SqgA, SQ C2-OH makes hydrogen bonding interactions with Tyr136 (2.6 Å), SQ C3-OH with His193 (2.7 Å) and Lys107 (2.6 Å), and no metal binding site is present.

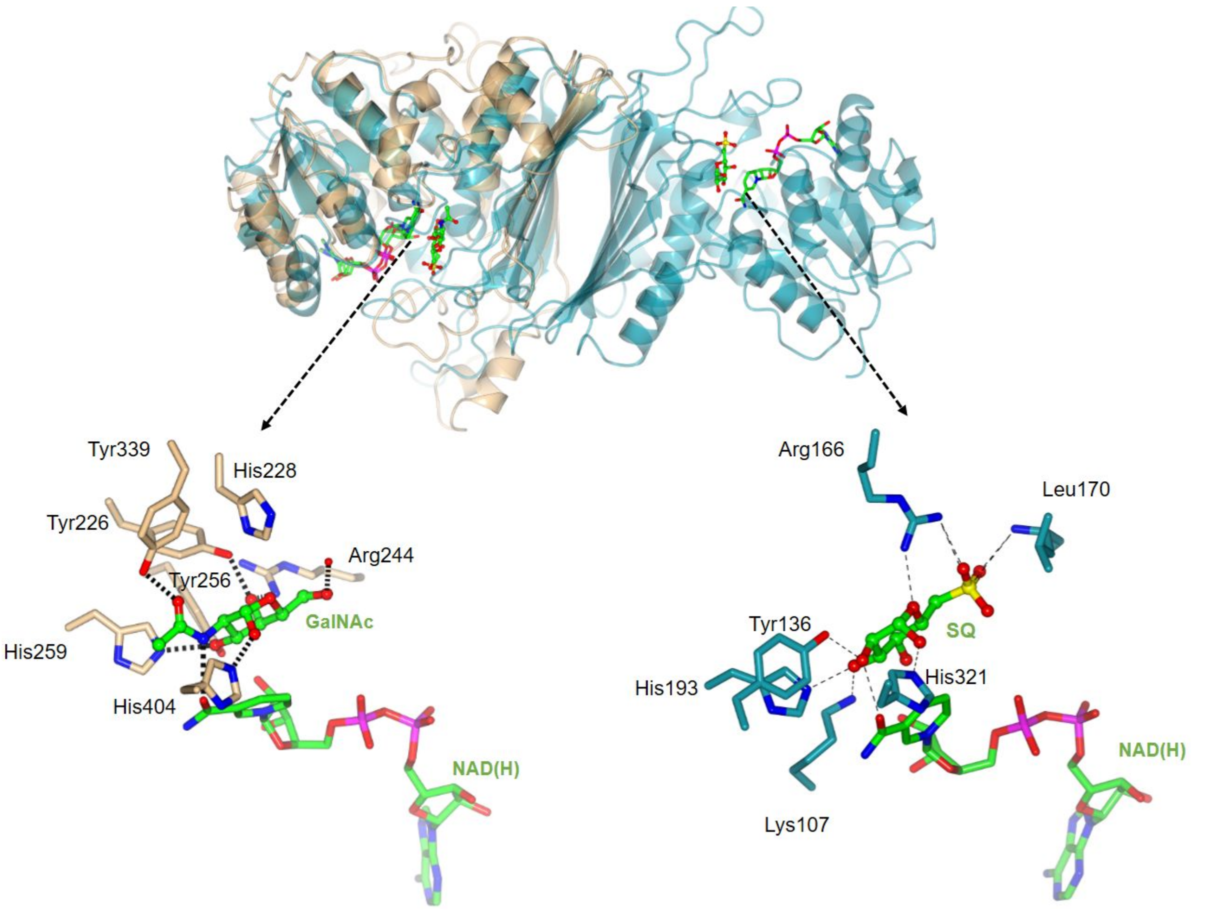

Figure S13. Superposition of structure of *Ar*SqgA•NAD$^+$•SQ (in dark cyan) with *Am*GH109A•NAD$^+$•GalNAc (PDB: 6T2B, in beige). Overlay reveals similar folds for both GH enzymes that use metal-independent, NAD$^+$- dependent redox mechanism s(RMS deviation of 2.15 Å over 293 aligned residues). Both proteins dimerise through interactions of flat β-sheet surface at the interface. In the *Am*GH109A•NAD$^+$•GalNAc ternary complex, His259 H-bonds C3-OH, and Tyr226 is 3.3 Å distant from C2 of GalNAc. For *Ar*SqgA, SQ C2-OH makes hydrogen bonding interactions with Tyr136 (2.6 Å), C3-OH with His193 (2.7 Å) and Lys107 (2.6 Å). No metal binding site is seen in either enzyme.

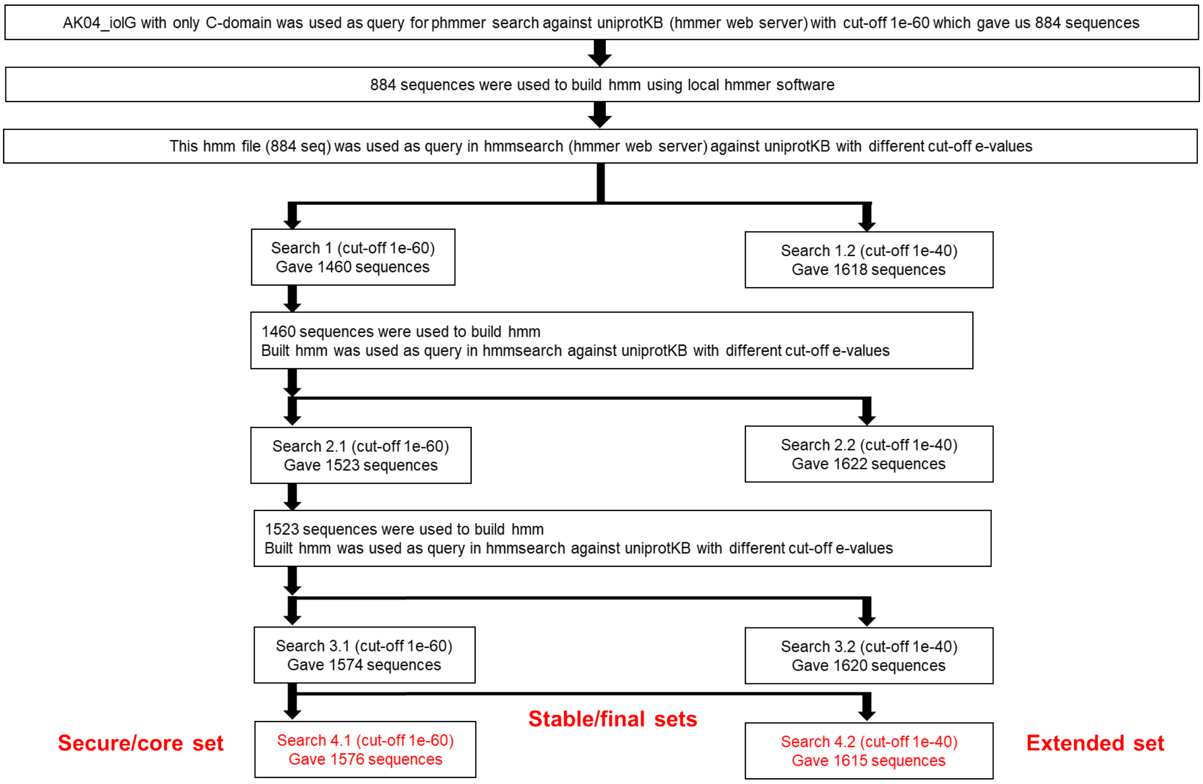

Figure S14. Hidden Markov model strategy for classification of new GH188 family.

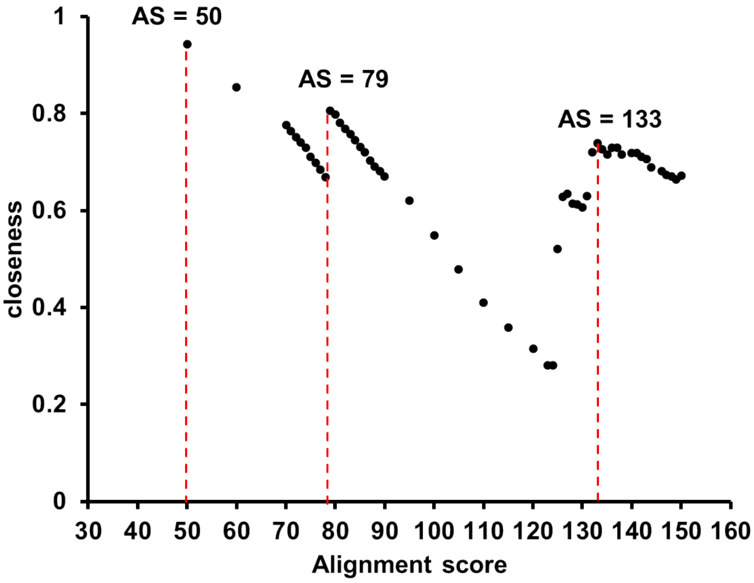

Figure S15. Plot of centrality closeness values against different alignment score of sequence similarity network.

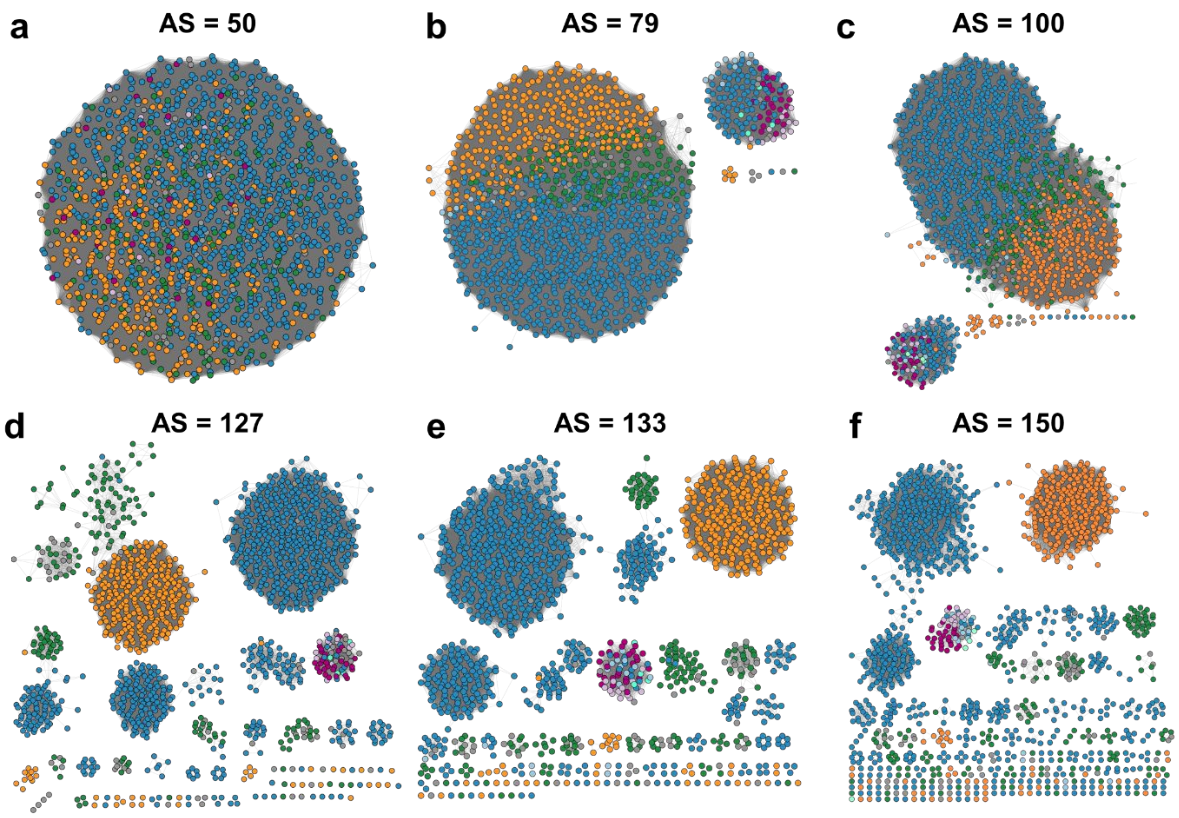

Figure S16. SSN of family GH188 members at different alignment scores. a) SSN at minimum alignment score 50, b) SSN at minimum alignment score 79, c) SSN at minimum alignment score 127, d) SSN at minimum alignment score 133. Nodes are colored according to taxonomy: Proteobacteria (blue), Firmicutes (pink), Actinobacteria (green), Deinococcus (light pink) Chloroflexi (light blue), Archaea (pale green), and Eukaryota (yellow).

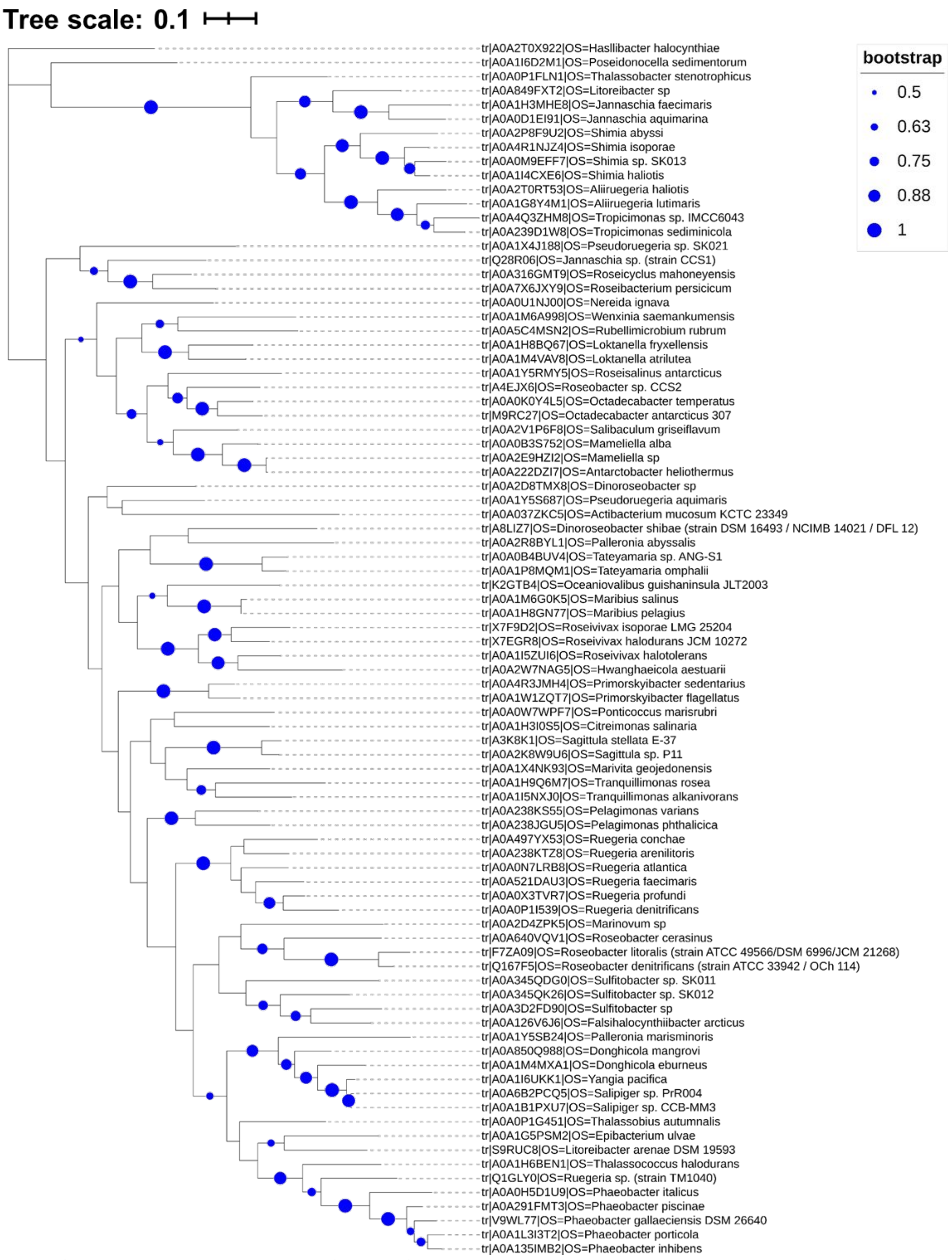

Figure S17. Phylogenetic tree of new GH188 family from *Roseobacter* clade bacteria. The phylogenetic tree of Roseobacteraceae family were constructed using MEGA (Molecular Evolutionary Genetics Analysis) software and formatted using iTOL (Interactive Tree of Life, https://itol.embl.de/). The size of blue circle at branches indicates the bootstrap proportion for 100 replicates.

Supplementary Tables

Table S1. Data collection and refinement statistics. Numbers in brackets refer to data for highest resolution shells.

	***Ar*SqgA•NAD⁺**	***Ar*SqgA•NAD⁺•SQ**	***Ar*SqgA•NAD⁺•citrate**
Data collection			
Space group	$P2_1$	$P2_12_12_1$	$P2_12_12_1$
Molecules in A.S.U	2	2	2
Cell dimensions			
a, *b*, *c* (Å)	73.27, 53.99, 90.72	56.56, 73.02, 199.50	56.86. 72.82, 197.31
α, β, ɣ (°)	90.00, 90.40, 90.00	90.00, 90.00, 90.00	90.00, 90.00, 90.00
Resolution (Å)	45.36-2.65 (2.78-2.65)	58.92-2.40 (2.49-2.40)	49.33-1.95 (2.00-1.95)
R_{merge}	0.153 (0.729)	0.270 (1.110)	0.167 (1.200)
R_{pim}	0.098 (0.463)	0.110 (0.470)	0.067 (0.475)
$I / \sigma I$	7.0 (2.2)	6.6 (2.1)	11.9 (2.5)
CC1/2	0.961 (0.709)	0.989 (0.723)	0.998 (0.911)
Completeness (%)	98.7 (98.6)	99.9 (99.7)	100.0 (100.0)
Redundancy	6.3 (6.6)	13.1 (11.9)	13.7 (14.2)
No. unique reflections	20601 (2715)	33269 (3423)	60807 (4220)
Refinement			
Resolution (Å)	45.30-2.65	58.8-2.40	49.30-1.95
R_{work} / R_{free}	0.2169/0.2748	0.1856/0.2438	0.1980/0.2386
No. atoms			
Protein	5420	5573	5533
Ligand/ion	88	118	114
Water	31	195	245
B-factors (Å^2)			
Protein	42	30	32
Ligand/ion	39	25	31
Water	26	26	33
R.m.s. deviations			
Bond lengths (Å)	0.0085	0.0091	0.0088
Bond angles (°)	1.50	1.45	1.45
Ramachandran Plot Residues			
In most favourable regions (%)	96.13	96.42	96.27
In allowed regions (%)	3.18	3.03	3.31
Outliers (%)	0.69	0.55	0.41
PDB code	**8QC3**	**8QC5**	**8QC6**

Table S2. Data collection and refinement statistics. Numbers in brackets refer to data for highest resolution shells.

	Fl**SqgA•NAD⁺**	***Fl*****SqgA•NAD⁺•SQ**
Data collection		
Space group	C 2 2 21	C 2 2 21
Molecules in A.S.U	4	4
Cell dimensions		
a, *b*, *c* (Å)	107.77, 161.49, 175.74	110.52, 160.38, 185.75
α, β, ɣ (°)	90.00, 90.00, 90.00	90.00, 90.00, 90.00
Resolution (Å)	59.45-2.35 (2.41-2.35)	51.19-2.30(2.35-2.30)
R_{merge}	0.186 (1.706)	0.281 (1.565)
R_{pim}	0.077 (0.806)	0.112 (0.617)
I / σI	8.0 (1.0)	6.2 (1.7)
CC1/2	0.998 (0.93)	0.995 (0.876)
Completeness (%)	99.7 (97.8)	100.0 (100.0)
Redundancy	13.0 (10.2)	13.8 (14.4)
No. unique reflections	63829 (4352)	73391 (4478)
Refinement		
Resolution (Å)	59.0-2.35	51.1-2.3
R_{work} / R_{free}	0.2285/0.2784	0.1950/0.2459
No. atoms		
Protein	10823	11072
Ligand/ion	206	284
Water	250	740
B-factors (Å²)		
Protein	57	33
Ligand/ion	46	29
Water	45	34
R.m.s. deviations		
Bond lengths (Å)	0.0086	0.0093
Bond angles (°)	1.43	1.48
Ramachandran Plot Residues		
In most favourable regions (%)	95.22	96.95
In allowed regions (%)	4.5	2.49
Outliers (%)	0.28	0.55
PDB code	**8QC8**	**8QC2**

Table S3. PFAM codes for neighborhood genes from different sulfoglycolytic and sulfolytic pathways.

Sr. No	PFAM	Protein	Pathways*
1.	PF01408, PF02894	SqgA Sulfoquinovosidase	sulfo-EMP1/2, sulfo-ED, sulfo-SFT, sulfo-SMO, sulfo-SDO
2.	PF01055	YihQ, SqvC Sulfoquinovosidase	sulfo-EMP1/2, sulfo-ED, sulfo-SFT, sulfo-SMO, sulfo-SDO, sulfo-TK
3.	PF01263	YihR SQ mutarotase	sulfo-EMP
4.	PF07221	YihS SQ-SF isomerase	sulfo-EMP, sulfo-SFT
5.	PF00294	YihV SF kinase	sulfo-EMP
6.	PF01791	YihT SFP aldolase	sulfo-EMP
7.	PF03446, PF14833	YihU SLA reductase	sulfo-EMP
8.	PF16161	SqvB SQ mutarotase	sulfo-EMP 2
9.	PF02952	SqvD SQ-SF isomerase	sulfo-EMP 2, sulfo-SFT
10.	PF00365	SqiK SF kinase	sulfo-EMP 2
11.	PF01116	SqiA SFP class II aldolase	sulfo-EMP 2
12.	PF02826	GabD/SlaB SLA dehydrogenase	sulfo-EMP 2, sulfo-ED, sulfo-SFT
13.	PF13561	SQ dehydrogenase	sulfo-ED
14.	PF08450	SGL lactonase	sulfo-ED
15.	PF00920	SG dehydratase	sulfo-ED
16.	PF01081, PF03328	KDSG aldolase	sulfo-ED
17.	PF00923	SftT, SqvA SF transaldolase	sulfo-SFT
18.	PF01613	SmoA, SsuE flavin reductase	sulfo-SMO, sulfo-ASMO
19.	PF00248	SmoB, SquF oxidoreductase	sulfo-SMO, sulfo-ASMO
20.	PF00296	SmoC, SquD SQ monooxygenase	sulfo-SMO, sulfo-ASMO
21.	PF02668	SqoD SQ dioxygenase	sulfo-SDO, sulfo-ASDO

* Sulfo-SMO = sulfo-ASMO; sulfo-SDO = sulfo-ASDO

Experimental Methods

1. General

Specialist reagents: SQ and methyl α-sulfoquinovoside were purchased from MCAT GmbH. $^{13}C_6$-SQ, 4-nitrophenyl α-D-sulfoquinovoside (α-PNPSQ), 4-nitrophenyl β-D-sulfoquinovoside (β-PNPSQ) and α-sulfoquinovosyl glycerol (SQGro) were synthesized as described.[1] 4-Nitrophenyl α-glucoside (α-PNPGlc), 4-nitrophenyl β-glucoside (β-PNPGlc), 4-nitrophenyl β-glucuronide (β-PNPGlcA) and all other reagents were purchased from Sigma-Aldrich.

Bacterial growth media: M9 media was prepared using M9 salt medium, trace metal solution and vitamin mixture. M9 salt medium consisted of 12.8 g/L Na_2HPO_4, 3 g/L KH_2PO_4, 1 g/L NH_4Cl, 0.5 g/L NaCl, 0.6 g/L $MgSO_4$, and 0.22 g/L $CaCl_2.2H_2O$. Trace metal solution consisted of 14.4 mg/L $FeCl_3$, 4.0 mg/L $CoCl_2.6H_2O$, 2.9 mg/L $CuCl_2.2H_2O$, 3.0 mg/L $MnCl_2.4H_2O$, 15.0 mg/L $ZnCl_2$, 0.2 mg/L $NiCl_2$, 3 mg/L $Na_2MoO_4.6H_2O$ and 6 mg/L H_3BO_3. Vitamin mixture consisted of 2 µg/L biotin, 5 µg/L calcium pantothenate, 10 µg/L thiamine hydrochloride, 10 µg/L p-aminobenzoic acid, 20 µg/L nicotinic acid, 50 µg/L pyridoxamine dihydrochloride, and 4 µg/L B12 (cyanocobalamin).

Growth studies: Cultures of *Arthrobacter* sp. AK01 were grown in M9 media, with glucose, MeSQ, SQ or SQGro (5 mM) as the sole carbon source. Cultures were incubated at 30 °C (250 rpm). Bacterial growth was monitored by using a custom built MicrobeMeter.[2] Growth experiments were replicated twice.

^{13}C NMR analysis of culture media: *Arthrobacter* sp. AK01 were grown to visible turbidity in M9 media containing 5 mM SQGro at 28 °C for 4 days with continuous shaking at 250 rpm. Cultures were centrifuged at 10000 rpm for 10 min (using Sigma laborzentrifugan model 1-15, rotor 12124) and the supernatant liquid was diluted to 50% D_2O. ^{13}C-NMR spectra of diluted supernatant liquid was recorded using a 500 MHz instrument.

2. Proteomics

Sample preparation for proteomic analysis: Frozen whole bacterial pellets were prepared using the in-StageTip preparation approach as previously described.[3] Cells were resuspended in 4% sodium deoxycholate (SDC), 100 mM Tris pH 8.0 and boiled at 95 °C with shaking for 10 min to aid solubilisation. Samples were allowed to cool for 10 minutes and then boiled for

a further 10 min before the protein concentration was determined by bicinchoninic acid assays (Thermo Fisher Scientific). 50 µg of protein for each biological replicate were reduced/alkylated with the addition of tris-2-carboxyethyl phosphine hydrochloride and iodoacetamide (final concentration 20 mM and 60 mM respectively), by incubating in the dark for 1 hour at 45 °C. Following reduction/alkylation samples were digested overnight with trypsin (1/25 w/w Solu-trypsin, Sigma) at 37 °C with shaking at 1000 rpm. Digests were halted by the addition of isopropanol and trifluoroacetic acid (50% and 1%, respectively) and samples cleaned up using home-made SDB-RPS StageTips prepared according to previously described protocols. [3-5] SDB-RPS StageTips were placed in a Spin96 tip holder[4] to enable batch-based spinning of samples and tips conditioned with 100% acetonitrile; followed by 30% methanol, 1% trifluoroacetic acid followed by 90% isopropanol, 1% trifluoroacetic acid with each wash spun through the column at 1000 x *g* for 3 min. Acidified isopropanol / peptide mixtures were loaded onto the SDB-RPS columns and spun through, and tips washed with 90% isopropanol, 1% trifluoroacetic acid followed by 1% trifluoroacetic acid in Milli-Q water. Peptide samples were eluted with 80% acetonitrile, 5% ammonium hydroxide and dried by vacuum centrifugation at room temperature, then were stored at -20 °C.

Reverse phase liquid chromatography–mass spectrometry: Prepared digested proteome samples were re-suspended in Buffer A* (2% acetonitrile, 0.01% trifluoroacetic acid) and separated using a two-column chromatography setup composed of a PepMap100 C_{18} 20-mm by 75-mm trap and a PepMap C_{18} 500-mm by 75-mm analytical column (Thermo Fisher Scientific). Samples were concentrated onto the trap column at 5 ml/min for 5 min with Buffer A (0.1% formic acid, 2% DMSO) and then infused into a Orbitrap Fusion Lumos equipped with a FAIMS Pro interface at 300 nl/min via the analytical columns using a Dionex Ultimate 3000 UPLCs (Thermo Fisher Scientific). 125-minute analytical runs were undertaken by altering the buffer composition from 2% Buffer B (0.1% formic acid, 77.9% acetonitrile, 2% DMSO) to 22% B over 95 min, then from 22% B to 40% B over 10 min, then from 40% B to 80% B over 5 min. The composition was held at 80% B for 5 min, and then dropped to 2% B over 2 min before being held at 2% B for another 8 min. The Fusion Lumos Mass Spectrometer was operated in a stepped high-field asymmetric-waveform ion mobility spectrometry (FAIMS) data-dependent mode at two different FAIMS compensation voltages (CVs) -40 and -60. For each FAIMS CV a single Orbitrap MS scan (300-1600 m/z and a resolution of 60k) was acquired every 1.7 seconds followed by Orbitrap MS/MS HCD scans of precursors (stepped NCE 25,35,45%, with a maximal injection time of 54 ms with the automatic gain control set to 250% and the resolution to 30k).

Proteomic data analysis: Identification and label-free quantification (LFQ) analysis were accomplished using MaxQuant (v1.6.17.0)[6] using the in-house generated proteome of AK01 allowing for oxidation on methionine. Prior to MaxQuant analysis, datasets acquired on the Fusion Lumos were separated into individual FAIMS fractions using the FAIMS MzXML Generator.[7] The LFQ and "Match Between Run" options were enabled to allow comparison between samples. The resulting data files were processed using Perseus (v1.4.0.6)[8] to compare the growth conditions using Student's t-tests as well as Pearson correlation analyses. For LFQ comparisons biological replicates were grouped and missing values imputed based on the observed total peptide intensities with a range of 0.3σ and a downshift of 1.8σ.

Data availability: The mass spectrometry proteomics data has been deposited in the Proteome Xchange Consortium via the PRIDE partner repository with the data set identifier: PXD043482 Username: reviewer_pxd043482@ebi.ac.uk Password: peKhCqz5

3. Amino acid sequences of targeted enzymes

>tr|A0A4R8X9U9|A0A4R8X9U9_9MICO Gfo/Idh/MocA family oxidoreductase OS=Cryobacterium sp. TMT2-4 OX=1259254 GN=E3O54_15865 PE=4 SV=1

MATIRYGIIGAGHMAREHVRNLALIPGSEITAIADPVEASREETSAAILGGEVHAFADYRDLLD
SDLVDAVIIASPNDTHHAILKDIFASGKAYPVLVEKPVCTTLADADELESLSRDYPAPVWVAM
EYRYMPPVQEILRTARNGDLGKLRMLSITEHRFPFLDKVGDWNRFSERTGGTLVEKCCHFF
DLMRLILQDEPVRIYASGARDVNHADERYDGRTPDILDNAYVVVDFAGGARAMLELCMFAE
GSLFQEHIAIVGDVAKTECYVPVAQGHWPLEDLREAEVEFSPRAPQAPRRHVVAVDDALLA
AGAHHGSTYYEHLGFRRAILGDGPVEVTVADGLQAVRMGIAAEQSIRDGRAVEIEPARIQVH
A

>tr|A0A1C9WRL0|A0A1C9WRL0_9MICC Oxidoreductase OS=Arthrobacter sp. U41 OX=1849032 GN=ASPU41_10185 PE=4 SV=1

MSLPTAEAVRTIRYGLIGAGHMAREHVRNLALIPGSLITAVSDPQPSSLEETVAEIGYEVTTF
PDHRELLVSGLVDALVIASPNDTHLDILKDIFSNQMKLPVLVEKPVCTTAAQADELESLAAGY
SAPVWVAMEYRYMPPVQELIQAAHGGKLGNVFMLSIVEHRFPFLHKVDAWNRFNERTGGT
LVEKCCHFFDLMRLILQDEPTRIYASGGHDVNHMDELYEGRVSDMIDNAYVVVDFKSGRRA
MLELSMFAEGSKFQERISIVGDAAKIECLIPVAASHWIEGDESEAVVEFSPRSPLGPETHEVP

VDEAVLAAGAHHGSTYYEHLGYRKAILGEGPVEVTVADGLQSVRMGLAAERSIIEGRPVELL
SANSAVS

>tr|A0A7D7VZ79|A0A7D7VZ79_FLASK Gfo/Idh/MocA family oxidoreductase OS=Flavobacterium sp. (strain K172) OX=37931 GN=FV140_09610 PE=4 SV=1

MSLPSGETAQTIRYGLIGAGHMAREHVRNLALIPGSLITAVADPTPSSLEETAREIGYDVQTF
SAHKDLLASGLVDALVIASPNDTHLEILKDIFASGTNLPVLVEKPVCTSAEQADELEELAATYT
APVWVAMEYRYMPPVQEIIQAAHSGKLGNIHMLSIVEHRFPFLHKVDAWNRFAERTGGTLV
EKCCHFFDLMRLILQDEPTRIYASGGHDVNHMDEVYDGLISDMVDNAYVIVDFKGGRRAML
ELSMFAEGSKFQERISIVGDAAKIETLIPVAANHWIEGDETEATVEFSPRSPLGPEKHEVPVD
EAVLAAGAHHGSTYYEHLGYRKAILGEGPVDVTVADGLQSVRMGLAAEQSIIEGRAVELHA
PSVAVQK

4. Preparation and analysis of SqgA enzymes

Cloning and expression: The vectors encoding *Fl*SqgA (UniProt: A0A7D7VZ79) and *Ar*SqgA UniProt: A0A1C9WRL0) were transformed into *E. coli* Rosetta(DE3) pLysS or BL21(DE3) cells (Novagen) and grown on Lysogeny Broth (LB) agar plates containing 50 μg mL^{-1} kanamycin for 16 hours at 37 °C. A single colony was picked and used to inoculate 10 mL of LB containing 50 μg mL^{-1} kanamycin and 30 μg mL^{-1} chloramphenicol. This pre-culture was incubated at 37 °C for 16 hours with shaking at 250 rpm, and then used to inoculate 1 L of LB containing 50 μg mL^{-1} kanamycin and 30 μg mL^{-1} chloramphenicol. The culture was incubated at 37 °C, with shaking at 250 rpm, until an OD_{600} of 0.6 was reached. The culture was cooled to 18 °C, then 0.5 mM isopropyl thiogalactoside was added and the culture incubated at 18 °C for a further 16 hours. For *Ar*SqgA, *E. coli* BL21 (DE3) cells were used without chloroamphenicol. The cells were harvested by centrifugation at 5000 x *g* for 20 min at 4 °C and the supernatant discarded. The pellet was re-suspended in 30 mL buffer A (50 mM Tris-HCl pH 7.5, 300 mM NaCl, 30 mM imidazole, 1 mM DTT) containing EDTA-free protease inhibitor cocktail (Roche cOmplete), 40 μg mL^{-1} lysozyme and 250 U benzonase nuclease (Sigma Aldrich). The cells were lysed by passage through a French Press (twice), operated at 25 kPsi, and soluble protein was isolated by centrifugation at 20000 x *g* for 40 minutes at 4 °C. The supernatant was loaded onto a pre-equilibrated HisTrap FF Crude 5 mL column (Cytiva) using an Äkta pure chromatography system (Cytiva). The column was washed with 5 column volumes (CV) of buffer A, then eluted with a gradient of buffer B (50 mM Tris-HCl pH 7.5, 300 mM NaCl, 500 mM imidazole, 1 mM DTT) in buffer A (0-100% B over 20 CV). SqgA containing fractions were

identified by SDS-PAGE, pooled and concentrated to a volume of 1 mL. The pooled fractions were loaded onto a pre-equilibrated HiLoad 16/600 200 pg gel filtration column (Cytiva) and proteins separated over 1 CV of buffer C (50 mM Tris-HCl pH 7.5, 300 mM NaCl, 1 mM DTT) using an Äkta pure chromatography system (Cytiva). SqgA containing fractions were identified by SDS-PAGE, pooled and concentrated.

SEC-MALLS analysis: Experiments were conducted on a system comprising a Wyatt HELEOS-II multi-angle light scattering detector and a Wyatt rEX refractive index detector linked to a Shimadzu HPLC system (SPD-20A UV detector, LC20-AD isocratic pump system, DGU-20A3 degasser and SIL-20A autosampler). Experiments were conducted at room temperature (20 ± 2 °C). Solvent was 0.2 μm filtered before use and a further 0.1 μm filter was present in the flow path. The column was equilibrated with at least 2 CV of buffer C before use and flow was continued at the working flow rate until baselines for UV, light scattering and refractive index detectors were all stable. 100 μL protein at a concentration of 2.5 mg mL^{-1} was injected. Shimadzu LabSolutions software was used to control the HPLC and Astra 7 software for the HELEOS-II and rEX detectors. The Astra data collection was 1 minute shorter than the LC solutions run to maintain synchronisation. Blank buffer injections were used as appropriate to check for carry-over between sample runs. Data were analysed using the Astra 7 software. Molecular weights were estimated using the Zimm fit method with degree 1. A value of 0.182 was used for protein refractive index increment (dn/dc).

5. Enzyme Characterization

Initial optimisation of reaction conditions:

a) pH profile of *Fl*SqgA: Reactions were conducted at 25 °C in 96-well plate format using 50 mM citrate/phosphate buffer (pH range 4.0-8.5), 20 mM NaCl, 1 mM NAD^+, 2 mM DTT, 0.1 mM $MnCl_2$, 12 mM α-PNPSQ and 1.86 μM of *Fl*SqgA in total volume of 100 μL. Reactions were monitored by measuring the absorbance at λ = 405 nm for the release of chromogenic 4-nitrophenolate ion as a product (extinction coefficient was 7830 M^{-1} cm^{-1} under the assay conditions). Absorbance was measured using Multimodal Plate Reader (FLUOstar omega, BMG Labtech). A concentration of α-PNPSQ of 12 mM was used, being $>K_M \times 10$. The Michaelis parameter k_{cat} was calculated for α-PNPSQ hydrolysis and plotted versus the pH range.

b) Optimisation of NAD^+ cofactor: Reactions were conducted at 25 °C in 96-well plate format in 50 mM Tris.HCl (pH 7), 20 mM NaCl, 0.1 mM $MnCl_2$, 10 mM α-PNPSQ and 1.86 μM *Fl*SqgA in total volume of 100 μL. The enzyme activity was measured in the presence of different cofactors i.e., NAD^+, $NADP^+$ and NADH at various concentrations i.e., 0.01, 0.1 and 1 mM. The Michaelis parameter k_{cat} was calculated for α-PNPSQ hydrolysis by monitoring the formation of product, 4-nitrophenolate ion.

Michaelis-Menten kinetics for *Fl*SqgA, *Ar*SqgA and *Cr*SqgA using α-PNPSQ and β-PNPSQ: Enzyme kinetics were measured for *Fl*SqgA, *Ar*SqgA and *Cr*SqgA catalysed hydrolysis of α-PNPSQ and β-PNPSQ. Release of chromogenic 4-nitrophenolate was monitored using a UV/visible spectrophotometer at 405 nm and an extinction coefficient of 7830 $M^{-1}cm^{-1}$ under the assay conditions. Reactions were conducted in 50 mM Tris.HCl (pH 7), 20 mM NaCl, 1 mM NAD^+, 2 mM DTT, 0.1 mM $MnCl_2$ at 25 °C using 1.86 μM to 2.24 μM of *Fl*SqgA, *Ar*SqgA and *Cr*SqgA at substrate concentrations ranging from 0.5 to 20 mM. Michaelis-Menten kinetic parameters (k_{cat}, K_M, k_{cat}/K_M) were calculated using Prism (GraphPad Scientific Software).

Michaelis-Menten activation analysis for *Fl*SqgA with NAD^+: Reaction rates were measured for *Fl*SqgA with constant concentration of α-PNPSQ and varied concentration of NAD^+. Release of chromogenic 4-nitrophenolate was monitored using a UV/visible spectrophotometer at 405 nm and an extinction coefficient of 7830 $M^{-1}cm^{-1}$ under the assay conditions. Reactions were conducted in 50 mM Tris.HCl (pH 7), 20 mM NaCl, 4 mM α-PNPSQ, 2 mM DTT, 0.1 mM $MnCl_2$ at 25 °C using 1.86 μM of *Fl*SqgA at NAD^+ concentrations ranging from 0.02 to 1.5 mM. The Michaelis-Menten activation constant (K_A) was calculated using Prism (GraphPad Scientific Software).

Michaelis-Menten kinetics for *Fl*SqgA using SQGro:

a) HPLC conditions for monitoring product formation: Formation of SQ upon cleavage of SQGro was measured using HPLC-ESI-MS/MS analysis. This used a triple quadrupole mass spectrometer (Agilent 6460 QQQ) coupled with Agilent 1260 Infinity Series LC system. The column was XBridge Premier BEH Amide VanGuard FIT Column, 2.5 μm, 4.6 mm x 150 mm. HPLC conditions were from 90% B to 20% B over 10 min; then 20% B for 5 min; back to 90% B in 2 min; 90% B for 10 min (Solvent A: 10 mM ammonium acetate in 1% acetonitrile; Solvent B: 100% acetonitrile); flow rate, 0.50 ml/min; injection volume, 5 μL. The mass spectrometer was operated in negative ionization mode. Quantification was achieved using MS/MS multiple reaction monitoring (MRM) mode of Agilent Mass Hunter Quantitative Analysis software and

normalized using α-PNPGlcA as internal standard. The sensitivity for each MRM-MS/MS transition was optimized for each analyte before analysis.

b) Measurement of reaction rates: A calibration curve for response to SQ was constructed using varying concentrations of SQ and a constant concentration of internal standard α-PNPGlcA. To demonstrate linearity of reaction rates, enzyme reactions for *Fl*SqgA were conducted in 100 µL volumes containing 50 mM Tris.HCl (pH 7), 20 mM NaCl, 1 mM NAD+, 2 mM DTT, 0.1 mM $MnCl_2$, 1 mM SQGro and 0.93 µM of VZ79. Reactions were initiated by addition of enzyme and incubated for 1 h at 30 °C. At 20, 40, 60 min time intervals 20 µL of reaction mixture was removed and quenched by heating at 80 °C for 4 min. The quenched reaction mixtures were mixed with 30 µL of internal standard (0.05 mM α-PNPGlcA) and analyzed by MS-MS.

c) Enzyme Kinetics: Enzyme assays were conducted in 100 µL volumes containing 50 mM Tris.HCl buffer (pH 7), 20 mM NaCl, 1 mM NAD^+, 2 mM DTT, 0.1 mM $MnCl_2$, and 0.93 µM of *Fl*SqgA and substrate concentrations ranging from 0.5 to 20 mM. Reactions were initiated by addition of enzyme and incubated for 30 min at 30 °C. After 20 min, the reaction was quenched by heating at 80 °C for 4 min. The quenched reaction mixtures were mixed with 30 µL of internal standard (i.e., 0.05 mM α-PNPGlcA) and analyzed by MS-MS.

Substrate specificity of *Fl*SqgA versus α- and β-PNPGlc and β-PNPGlcA: Specificity of *Fl*SqgA was tested against α- and β-PNPGlc and β-PNPGlcA using 100 µL reaction mixtures containing 50 mM Tris.HCl buffer (pH 7), 20 mM NaCl, 1 mM NAD^+, 2 mM DTT, 0.1 mM $MnCl_2$ and 10 mM α- or β-PNPGlc or β-PNPGlcA at 25 °C using 1.86 µM of *Fl*SqgA. Reactions were monitored continuously for 1 h by recording the absorbance at λ = 405 nm for the release of chromogenic 4-nitrophenolate ion as a product (extinction coefficient was 7830 M^{-1} cm^{-1} under the assay conditions). Absorbance was measured using Multimodal Plate Reader (FLUOstar omega, BMG Labtech).

Deuterium labelling experiments: Buffers and reagents were prepared in 99.9% D_2O. *Fl*SqgA was exchanged into deuterated buffer solutions by dialysis using slide-A-lyzer mini dialysis with a nominal molecular weight limit (NMWL) of 10 kDa. Reactions were conducted at 25 °C in 50 mM Tris.HCl (pH 7), 20 mM NaCl, 0.1 mM $MnCl_2$, 25 mM α-PNPSQ and 2.24 µM *Fl*SqgA in a total volume of 1000 mL. The reaction mixture was incubated at 25 °C for overnight and product formation was measured using HPLC-ESI-MS/MS analysis using a triple quadrupole mass spectrometer (Agilent 6460 QQQ) coupled with Agilent 1260 Infinity Series LC system. The column was XBridge Premier BEH Amide VanGuard FIT Column, 2.5

µm, 4.6 mm x 150 mm. HPLC conditions were from 90% B to 20% B over 10 min; then 20% B for 5 min; back to 90% B in 2 min (Solvent A: 10 mM ammonium acetate in 1% acetonitrile; Solvent B: 100% acetonitrile); flow rate, 0.50 ml/min; injection volume, 5 µL. The total-ion chromatograms (TICs) and product ion scan for reaction mixture and standard SQ was recorded in the negative-ion mode. The retention times and ESI–MS–MS fragmentation patterns of the analytes were observed as follows:

SQ retention time, 8.1 min; SQ ESI–MS *m/z* (% base-peak) 243; SQ ESI–MS–MS of $[M-H]^-$ 243: 207, 183, 153, 123, 81.

D-SQ retention time, 8.1 min; D-SQ ESI–MS *m/z* (% base-peak) 244.1; D-SQ ESI–MS–MS of $[M-H]^-$ 244.1: 207, 183, 153, 123, 81.

6. Protein Crystallisation

Crystallization of *Fl*SqgA: Crystals were grown in optimised MRC maxi 48-well plates (Swissci) using the sitting-drop vapour-diffusion method at 20 °C. 0.16 µL of a 15 mg/mL protein solution in buffer C (50 mM Tris-HCl pH 7.5, 300 mM NaCl, 1 mM DTT) was dispensed with 0.14 µL of mother liquor for *Fl*SqgA crystals.

*Fl*SqgA crystals were formed in wells containing 100 mM Bis-tris propane pH 7, 0.02 mM sodium and potassium phosphate, 28% (w/v) PEG 3350, 5 mM NAD^+ and 1 mM manganese chloride after 2 months. For the *Fl*SqgA•NAD^+•SQ complex, SQ powder was soaked into the well directly for around 2 min.

Crystallization of *Ar*SqgA: Crystals were grown in commercially available INDEX (Hampton Research) screens in 96-well sitting drop trays using the sitting-drop vapour-diffusion method at 20 °C. 0.15 µL of a 6 mg/mL protein solution for the *Ar*SqgA•NAD+•citrate complex, and 14 mg/mL for the *Ar*SqgA•NAD^+ and *Ar*SqgA•NAD^+•SQ complexes, were dispensed in buffer C (50 mM Tris-HCl pH 7.5, 300 mM NaCl, 1 mM DTT) with 0.15 µL mother liquor.

Crystals that provided the *Ar*SqgA•NAD^+•CIT complex were formed in wells containing 1.8 M triammonium citrate pH7 and 2 mM NAD^+ (SQ powder was soaked into this crystal but density for the sugar was not observed). For the *Ar*SqgA•NAD^+ complex, crystals were formed in wells containing 0.2 M potassium nitrate pH 6.8, 20% (w/v) PEG 3350, 2 mM NAD^+. For the *Ar*SqgA•NAD^+•SQ complex, the protein was combined with 10 mM SQ for 30 min prior to

setting up the crystallization plate and crystals were formed in wells containing 25% PEG 1500 (w/v).

Crystals were harvested using nylon CryoLoops (Hampton Research) and cryopreserved in liquid nitrogen without cryoprotectant.

X-ray data collection: Diffraction data were collected at Diamond light source, Didcot, Oxfordshire, UK (at I03 beamlines).The data were processed and integrated using XDS[9] and scaled using SCALA[10] included in the Xia2 processing system.[11] Data collection and refinement statistics are given in Supplementary Tables S1-2. The structures were solved using MOLREP,[12] employing the alphafold model AF-A0A7D7VZ79-F1[13, 14] (found using MrParse).[15] The structure was built and refined using iterative cycles using Coot [16] and REFMAC,[17] the latter employing local NCS restraints. Following building and refinement of the protein and water molecules, clear residual density was observed in the omit maps for ligands. The coordinate and refinement library files for SQ were prepared using ACEDRG.[18] Library files for NAD^+ were obtained from the COOT dictionary. SQ was modelled at occupancy of 0.8 in *Fl*SqgA•NAD^+•SQ structure and at occupancy of 1 in *Ar*SqgA•NAD^+•SQ; all other ligands/ions were modelled at occupancy of 1.

Structure analyses: All structure figures were generated using ccp4mg. The protein interactions, surfaces, and assemblies (PISA) server[11] was used to deduce the dimerization interface and buried surface area. Crystal packing interactions were analyzed using PISA.[12] Structural comparisons and structure-based sequence alignments were conducted using a DALI search of the Protein Data Bank[13].

7. Bioinformatics

Family classification of newly identified sulfoquinovosidase: The C-terminal domain of protein sequence (SqgA) from *Arthrobacter* sp. strain AK01 were used as query in phmmer web server (https://www.ebi.ac.uk/Tools/hmmer/search/phmmer).[19] Standard parameters were used except the cut-off for *E*-value was set to 10^{-60}. The resulting sequences were used to build a hidden Markov model (HMM), which was used as query for *hmmsearch* in hmmer web server (https://www.ebi.ac.uk/Tools/hmmer/search/hmmsearch) with two different cut-off *E*-values i.e., 10^{-60} and 10^{-40}. The retrieved sequences were used to build a HMM and used as query for hmmsearch with different cut-off values. This step was repeated until the stable number of sequences were retrieved.

Subfamily classification of SQase: The finalised set of new GH family was used to generate sequence similarity networks using the Enzyme Function Initiative-Enzyme Similarity Tool (https://efi.igb.illinois.edu/efi-est/)[20, 21] with range of alignment scores from 50 to 160. The generated SSNs were used to calculate the average closeness centrality values as described.[22] The centrality values were plotted against the alignment score.

Sequence similarity network analysis: Sequence similarity networks using the Enzyme Function Initiative-Enzyme Similarity Tool (https://efi.igb.illinois.edu/efi-est/) were generated for finalised set of family. The SSN generated from the alignment score 133 were used to generate genome neighborhood diagrams with open reading frame ± 10 neighbours using the Enzyme Function Initiative-Genome Neighbourhood Tool (https://efi.igb.illinois.edu/efi-gnt/). The individual nodes of the SqgA SSN were manually colored according to the occurrence of gene homologues (PFAM) from various pathways in their neighborhood (i.e., sulfo-EMP, sulfo-ED, sulfo-SFT, sulfo-SMO, sulfo-TK) using Cytoscape.[23] The nodes were selected based on the PFAM of neighborhood gene homologues from sulfoglycolytic and sulfolytic pathways in their neighborhood and colored depending on their pathways i.e., sulfo-EMP as green nodes sulfo-ED as pink nodes, sulfo-SFT as brown nodes, sulfo-SMO as blue nodes and sulfo-SDO as light purple. To display the taxonomic distribution of SqgA, the SSN was manually colored based on their phylum.

Supporting References

1. Abayakoon, P.; Epa, R.; Petricevic, M.; Bengt, C.; Mui, J. W. Y.; van der Peet, P. L.; Zhang, Y.; Lingford, J. P.; White, J. M.; Goddard-Borger, E. D.; Williams, S. J., Comprehensive synthesis of substrates, intermediates and products of the sulfoglycolytic Embden-Meyerhoff-Parnas pathway. *J. Org. Chem.* **2019,** *84*, 2901-2910.
2. Sasidharan, K.; Martinez-Vernon, A. S.; Chen, J.; Fu, T.; Soyer, O. S., A low-cost DIY device for high resolution, continuous measurement of microbial growth dynamics. *bioRxiv* **2018**, 407742.
3. Kulak, N. A.; Pichler, G.; Paron, I.; Nagaraj, N.; Mann, M., Minimal, encapsulated proteomic-sample processing applied to copy-number estimation in eukaryotic cells. *Nat. Methods* **2014,** *11*, 319-24.
4. Harney, D. J.; Hutchison, A. T.; Hatchwell, L.; Humphrey, S. J.; James, D. E.; Hocking, S.; Heilbronn, L. K.; Larance, M., Proteomic Analysis of Human Plasma during Intermittent Fasting. *J. Proteome Res.* **2019,** *18*, 2228-2240.
5. Rappsilber, J.; Mann, M.; Ishihama, Y., Protocol for micro-purification, enrichment, pre-fractionation and storage of peptides for proteomics using StageTips. *Nat. Protoc.* **2007,** *2*, 1896-1906.
6. Cox, J.; Mann, M., MaxQuant enables high peptide identification rates, individualized p.p.b.-range mass accuracies and proteome-wide protein quantification. *Nat. Biotechnol.* **2008,** *26*, 1367-1372.
7. Hebert, A. S.; Prasad, S.; Belford, M. W.; Bailey, D. J.; McAlister, G. C.; Abbatiello, S. E.; Huguet, R.; Wouters, E. R.; Dunyach, J.-J.; Brademan, D. R.; Westphall,

M. S.; Coon, J. J., Comprehensive single-shot proteomics with FAIMS on a hybrid Orbitrap mass spectrometer. *Anal. Chem.* **2018,** *90*, 9529-9537.
8. Tyanova, S.; Temu, T.; Sinitcyn, P.; Carlson, A.; Hein, M. Y.; Geiger, T.; Mann, M.; Cox, J., The Perseus computational platform for comprehensive analysis of (prote)omics data. *Nat. Methods* **2016,** *13*, 731.
9. Kabsch, W., Xds. *Acta Crystallogr., Section D: Biol. Crystallogr.* **2010,** *66*, 125-132.
10. Evans, P., Scaling and assessment of data quality. *Acta Crystallogr. Sect. D* **2006,** *62*, 72-82.
11. Winter, G., xia2: an expert system for macromolecular crystallography data reduction. *J. Appl. Crystallogr.* **2010,** *43*, 186-190.
12. Vagin, A.; Teplyakov, A., MOLREP: an Automated Program for Molecular Replacement. *J. Appl. Crystallogr.* **1997,** *30*, 1022-1025.
13. Jumper, J.; Evans, R.; Pritzel, A.; Green, T.; Figurnov, M.; Ronneberger, O.; Tunyasuvunakool, K.; Bates, R.; Žídek, A.; Potapenko, A.; Bridgland, A.; Meyer, C.; Kohl, S. A. A.; Ballard, A. J.; Cowie, A.; Romera-Paredes, B.; Nikolov, S.; Jain, R.; Adler, J.; Back, T.; Petersen, S.; Reiman, D.; Clancy, E.; Zielinski, M.; Steinegger, M.; Pacholska, M.; Berghammer, T.; Bodenstein, S.; Silver, D.; Vinyals, O.; Senior, A. W.; Kavukcuoglu, K.; Kohli, P.; Hassabis, D., Highly accurate protein structure prediction with AlphaFold. *Nature* **2021,** *596*, 583-589.
14. Varadi, M.; Anyango, S.; Deshpande, M.; Nair, S.; Natassia, C.; Yordanova, G.; Yuan, D.; Stroe, O.; Wood, G.; Laydon, A.; Žídek, A.; Green, T.; Tunyasuvunakool, K.; Petersen, S.; Jumper, J.; Clancy, E.; Green, R.; Vora, A.; Lutfi, M.; Figurnov, M.; Cowie, A.; Hobbs, N.; Kohli, P.; Kleywegt, G.; Birney, E.; Hassabis, D.; Velankar, S., AlphaFold Protein Structure Database: massively expanding the structural coverage of protein-sequence space with high-accuracy models. *Nucleic Acids Res.* **2022,** *50*, D439-D444.
15. Simpkin, A. J.; Thomas, J. M. H.; Keegan, R. M.; Rigden, D. J., MrParse: finding homologues in the PDB and the EBI AlphaFold database for molecular replacement and more. *Acta Crystallogr. Sect. D* **2022,** *78*, 553-559.
16. Emsley, P.; Cowtan, K., Coot: Model-building tools for molecular graphics. *Acta Crystallogr., Sect. D: Biol. Crystallogr.* **2004,** *60*, 2126-2132.
17. Murshudov, G. N.; Vagin, A. A.; Dodson, E. J., Refinement of Macromolecular Structures by the Maximum-Likelihood Method. *Acta Crystallogr. Sect. D* **1997,** *53*, 240-255.
18. Long, F.; Nicholls, R. A.; Emsley, P.; Grazulis, S.; Merkys, A.; Vaitkus, A.; Murshudov, G. N., AceDRG: a stereochemical description generator for ligands. *Acta Crystallogr. Sect. D* **2017,** *73*, 112-122.
19. Potter, S. C.; Luciani, A.; Eddy, S. R.; Park, Y.; Lopez, R.; Finn, R. D., HMMER web server: 2018 update. *Nucleic Acids Res.* **2018,** *46*, W200-W204.
20. Zallot, R.; Oberg, N.; Gerlt, J. A., The EFI web resource for genomic enzymology tools: leveraging protein, genome, and metagenome databases to discover novel enzymes and metabolic pathways. *Biochemistry* **2019,** *58*, 4169-4182.
21. Oberg, N.; Zallot, R.; Gerlt, J. A., EFI-EST, EFI-GNT, and EFI-CGFP: Enzyme Function Initiative (EFI) Web Resource for Genomic Enzymology Tools. *J. Mol. Biol.* **2023,** *435*, 168018.
22. Hornung, B. V. H.; Terrapon, N., An objective criterion to evaluate sequence-similarity networks helps in dividing the protein family sequence space. *bioRxiv* **2022**, 2022.04.19.488343.
23. Shannon, P.; Markiel, A.; Ozier, O.; Baliga, N. S.; Wang, J. T.; Ramage, D.; Amin, N.; Schwikowski, B.; Ideker, T., Cytoscape: a software environment for integrated models of biomolecular interaction networks. *Genome Res.* **2003,** *13*, 2498-504.
